# Integrative Predictive Modeling of Metastasis in Melanoma Cancer Based on MicroRNA, mRNA, and DNA Methylation Data

**DOI:** 10.3389/fmolb.2021.637355

**Published:** 2021-09-23

**Authors:** Ayşegül Kutlay, Yeşim Aydin Son

**Affiliations:** Department of Health Informatics, Graduate School of Informatics, METU, Ankara, Turkey

**Keywords:** machine learning, melanoma, metastasis, metastatic molecular signatures, miRNA, mRNA, DNA methylation

## Abstract

**Introduction:** Despite the significant progress in understanding cancer biology, the deduction of metastasis is still a challenge in the clinic. Transcriptional regulation is one of the critical mechanisms underlying cancer development. Even though mRNA, microRNA, and DNA methylation mechanisms have a crucial impact on the metastatic outcome, there are no comprehensive data mining models that combine all transcriptional regulation aspects for metastasis prediction. This study focused on identifying the regulatory impact of genetic biomarkers for monitoring metastatic molecular signatures of melanoma by investigating the consolidated effect of miRNA, mRNA, and DNA methylation.

**Method:** We developed multiple machine learning models to distinguish the metastasis by integrating miRNA, mRNA, and DNA methylation markers. We used the TCGA melanoma dataset to differentiate between metastatic melanoma samples by assessing a set of predictive models. For this purpose, machine learning models using a support vector machine with different kernels, artificial neural networks, random forests, AdaBoost, and Naïve Bayes are compared. An iterative combination of differentially expressed miRNA, mRNA, and methylation signatures is used as a candidate marker to reveal each new biomarker category’s impact. In each iteration, the performances of the combined models are calculated. During all comparisons, the choice of the feature selection method and under and oversampling approaches are analyzed. Selected biomarkers of the highest performing models are further analyzed for the biological interpretation of functional enrichment.

**Results:** In the initial model, miRNA biomarkers can identify metastatic melanoma with an 81% F-score. The addition of mRNA markers upon miRNA increased the F-score to 92%. In the final integrated model, the addition of the methylation data resulted in a similar F-score of 92% but produced a stable model with low variance across multiple trials.

**Conclusion:** Our results support the role of miRNA regulation in metastatic melanoma as miRNA markers model metastasis outcomes with high accuracy. Moreover, the integrated evaluation of miRNA with mRNA and methylation biomarkers increases the model’s power. It populates selected biomarkers on the metastasis-associated pathways of melanoma, such as the “osteoclast”, “Rap1 signaling”, and “chemokine signaling” pathways.

**Source Code:** https://github.com/aysegul-kt/MelonomaMetastasisPrediction/

## Introduction

Melanoma, a cancer with a rapid increase in incidence and high mortality, is a malignant tumor of skin pigmentation cells with a high mortality rate. Melanoma can develop anywhere on the body but is most commonly observed in areas exposed to the sun, such as the back, legs, arms, and face. With nearly 300,000 cases, melanoma is one of the most common cancer types worldwide (World Cancer Research Fun, 2021).

According to CDC statistics, 85,000 new cases are reported in the United States on a yearly basis, where 8,000 people die annually (United States Cancer Stat, 2021). In the European Union, on the other hand, melanoma cancer incidence reaches 14,000 annual cases. It is considered one of the fastest rising forms of cancer, albeit with hot spots in Europe, those being Scandinavian countries, Switzerland, and Austria (American Cancer Society, 2016). In addition, 16,000 new melanoma cases have been reported in the United Kingdom, which corresponds to 4% of all cancer types, and it has had a rising incidence rate of 135% over 30 years (Cancer Research UK, 2017).

Both distant and regional metastases are possible in melanomas. The most common metastasis sites in melanoma cases are bone, the brain, the liver, the lung, and skin. The presence of skin metastasis may be the first outward sign of lymphatic or hematogenous spreading. So in melanoma, rather than diagnosis, the prognosis is a critical concern. It is possible to detect at least suspicious cases *via* visual examination or short screening. Early diagnosis leads to high cure rates, but there is still no effective treatment in later stages, where metastasis is observed frequently (Damsky et al., 2010).

### Signatures for Metastasis

When the balance between cell growth and death is disrupted as a result of either “uncontrolled cell growth” or “loss of apoptosis (programmed cell death)”, tumorigenesis starts (Ma and Weinberg, 2008; Oppenheimer, 2006). At the initial stage, a malignant tumor presents at the site of the initial conversion of a normal cell to a tumor cell, called a primary tumor. This primary tumor may stay stable in this originated tissue (benign) or spread to the other parts of the body (malignant) by invasion or metastasis (Carter, 1974; Oppenheimer, 1982; Tonini et al., 2003; Shen et al., 2013). Understanding the molecular basis of carcinogenesis is essential in preventing, diagnosing, and treating cancer and its metastasis (Harris, 1991).

Many different markers have been proposed to describe the molecular foundation of metastasis. DNA methylation, gene expression profiles, and microRNAs are frequent biomarkers for predicting metastasis for most cancer types.

MicroRNAs are noncoding RNAs and regulate proliferation, cell cycle control, apoptosis, differentiation, migration, and metabolism (Kasinski and Slack, 2011; Jansson and Lund, 2020; Stahlhut and Slack, 2013). So, it is not surprising that microRNAs play a crucial role as suppressors or promoters of carcinogenesis or metastasis by controlling their target mRNA (Shalaby et al., 2014). Based on this understanding, microRNAs became the main focus in cancer biology and were proven to be crucial components of the normal and pathologic states of cells (Stahlhut and Slack, 2013; Hayes et al., 2014).

DNA methylation is a chemical process in which DNA binds with a methyl group. This process modifies the functionality of the DNA itself. It is an important regulator that plays a crucial role in genomic imprinting, X-chromosome inactivation, repression of repetitive elements, and aging. DNA methylation associates with many types of cancer (Zhang et al., 2011). Global hypomethylation also implicates cancer development and progression through different mechanisms (Craig and Wong, 2011). Typically, there is hypermethylation of tumor suppressor genes and hypomethylation of oncogenes (Gonzalo, 2020; Melchers et al., 2015).

### Predictive Models for Metastasis for Other Cancer Types

Although predictive machine learning models for melanoma metastasis are limited, many studies propose predictive biomarkers for different metastatic cancers. While most of the studies target specific markers such as microRNA or protein expression, recent studies (Souza et al., 2017) investigate the integrated usage of miRNA and mRNA signatures.

Binary logistic regression, which uses miRNA-331 and miRNA-195 as markers, is able to distinguish between metastasis and local breast cancer (sensitivity = 0.95 and specificity = 0.76) (McAnena et al., 2019). A study conducted by Souza et al. (2017) developed an integrated model using the expression levels of 27 miRNAs and 81 target mRNAs to classify prostate cancer patients from controls with 67% sensitivity and 75% specificity. Another study reports a statistical model with 71.4% accuracy for forecasting lymph node metastasis with independent test cases (Moriya et al., 2009). Besides, the SVM (support vector machine) classifier, which uses gene expression profiling with microarrays, predicts metastasis with 78% accuracy for breast cancer (Burton et al., 2012). To predict the lymph node metastasis of primary lung cancer tumors, computerized tomography (CT) and mRNA expression profiling are combined *via* statistical analysis (Chang et al., 2008). This method increased the accuracy from 55% (CT) to 86% (CT and mRNA). A statistical model built with ANOVA and hierarchical clustering predicts future metastasis in head and neck squamous cell carcinoma (HNSCC) with an accuracy of 77% (Rickman et al., 2008). The research conducted by Kan et al. (2004) proposed a predictive model for “lymph node metastasis” by using artificial neural networks based on gene expression profiles of primary tumors with an accuracy of 77%.

Chen and colleagues (Chen et al., 2009) studied “cancer metastasis networks”. In that study, a large set of patient data and the prediction of progression patterns generated a system network for the primary tumor and the sides of metastasis. By using these networks (which are constructed by hierarchical clustering), they have tried to predict the primary site of the tumor after a sequence of metastasis multinomial logistic regression with an overall accuracy of 51% (prostate, 84%; colon, 80%; lung and bronchus, 69%; ovary, 64%; larynx, 61%; and female breast, 56%).

Roessler et al. (2010) have generated a risk classifier tool to predict hepatocellular tumors by using gene expression levels with a combination of serum AFP levels or BCLC staging. Among six different prediction algorithms—support vector machines (SVMs), nearest centroid (NC), 3-nearest neighbor (3-NN), 1-nearest neighbor (1-NN), linear discriminant analysis (LDA), or compound covariate predictor (CCP)—CCP achieved the best sensitivity and specificity (76 and 60%, respectively) on cases from the “Liver Cancer Institute”. They also tested the model on another case set from the “Laboratory of Experimental Carcinogenesis”. The model predicts the risk with a sensitivity of 84% and a specificity of 65%.

Another study (Watanabe et al., 2010) proposed a model to predict liver metastasis with a primary colorectal tumor by using gene expression profiles of DNA microarray samples with the k-nearest neighbor (KNN) method and 10-fold cross-validation. The model predicts metastasis with 86.2% accuracy. Zemmour et al. (2015) developed three models (elastic net, LASSO, and CoxBoost) to predict early breast cancer metastasis using DNA microarray data. The study used a publicly available dataset as a training set. Then they validated the results on two different datasets (van de Vijver’s and Desmedt’s). The model predicts metastasis with 66% accuracy on the previous and 59% accuracy on the other dataset.

### Predictive Models for Melanoma Metastasis

Unlike other cancers, there are limited studies on modeling melanoma metastasis. Recently, serum levels of the cytokines IL‐4, GM‐CSF, and DCD and the Breslow thickness were proposed as markers to predict melanoma metastasis, where a linear regression achieved the best balance accuracy (80%) in the test set (Mancuso et al., 2020). A deep convolutional neural network (DCNN) study to predict BAP1 mutation also identified decisive prognostic factors for predicting metastatic risk *via* whole slide images with an area under the curve of 0.90 (Zhang et al., 2020). Additionally, mir-205-5p was found to be a significant biomarker for metastatic melanoma by Valentini et al. (2019). Also, Wei et al. (2019) indicated that TRIM44-tripartite motif-containing protein-44, regulated by miR-26b-5p, was identified as amplified on melanoma tissues. The same study reported miR-26-5p as downregulated on melanoma. The study conducted by Kinslechner et al. (2019) showed that scavenger receptor class B type 1 (SR-BI) protein expression contributes to metastatic melanoma. Wang et al. (2019) proposed long noncoding RNA TUG1 as a prognostic biomarker of metastatic melanoma. Besides, they have also indicated that miR-29c-3p, which is the target for G-protein signaling 1 (RGS1), suppresses the expression of TUG1.

Overall, transcriptional regulation is one of the critical mechanisms underlying cancer development. Even though mRNA, microRNA, and DNA methylation mechanisms have a critical impact on metastatic outcomes, there are no comprehensive data mining models that combine all aspects of transcriptional regulation for metastasis prediction. In this study, we focused on identifying the regulatory impact of genetic biomarkers for monitoring metastatic molecular signatures of melanoma by investigating the consolidated effect of miRNA, mRNA, and DNA methylation. We used differentially expressed miRNA, mRNA, and methylation signatures on the TCGA melanoma dataset to distinguish metastatic melanoma samples by assessing a set of predictive models. The highest performing model is selected, and its biomarkers are further analyzed for the biological interpretation of functional enrichment and to determine regulatory networks.

We used the TCGA Skin Cutaneous Melanoma (SKCM) dataset, which has been analyzed in various studies on the overall survival and identification of prognostic markers based on genomics data. Xiong et al. (2019) used clinical data and miRNA sequencing data to associate the observed survival rate. Similarly, Chen et al. (2017), Yang et al. (2018), Ma et al. (2017), and Xue et al. (2020) studied RNA sequencing data and proposed noncoding RNAs for SKCM prognosis. Guo et al. (2015) combined miRNA and mRNA sequencing data and proposed 15 miRNAs and 5 mRNAs for prognosis. Additionally, Jiang et al. presented the integration of mutation, copy number variation, methylation, and mRNA expression data for identifying prognostic markers. Selitsky et al. (2019) used mRNA sequencing data and applied machine learning models to measure the relative similarity of gene expression profiles of bulk tumor samples and different B cell phenotypes.

## Materials and Methods

In the study, opened data for Skin Melanoma (SKCM) (The Cancer Genome Atlas N, 2015) of the TCGA (The Cancer Genome Atlas) database are used, which is a part of the TCGA dataset served on the Cancer Genomics Cloud (CGC). The Cancer Genomics Cloud (CGC) (Institute, 2020) hosts a large genomic dataset and provides tools for searching and analyzing genomic data, serving as a computational environment on the cloud. The data browser tool provided by the CGC is used to search for TCGA cases and CCLE cell lines. On TCGA, a melanoma dataset with 470 cases composed of 352 metastatic and 97 primary tumor samples is used during this study, with three experimental strategies in the dataset, namely, miRNA expression, mRNA expression, and methylation.

**FIGURE 1:**
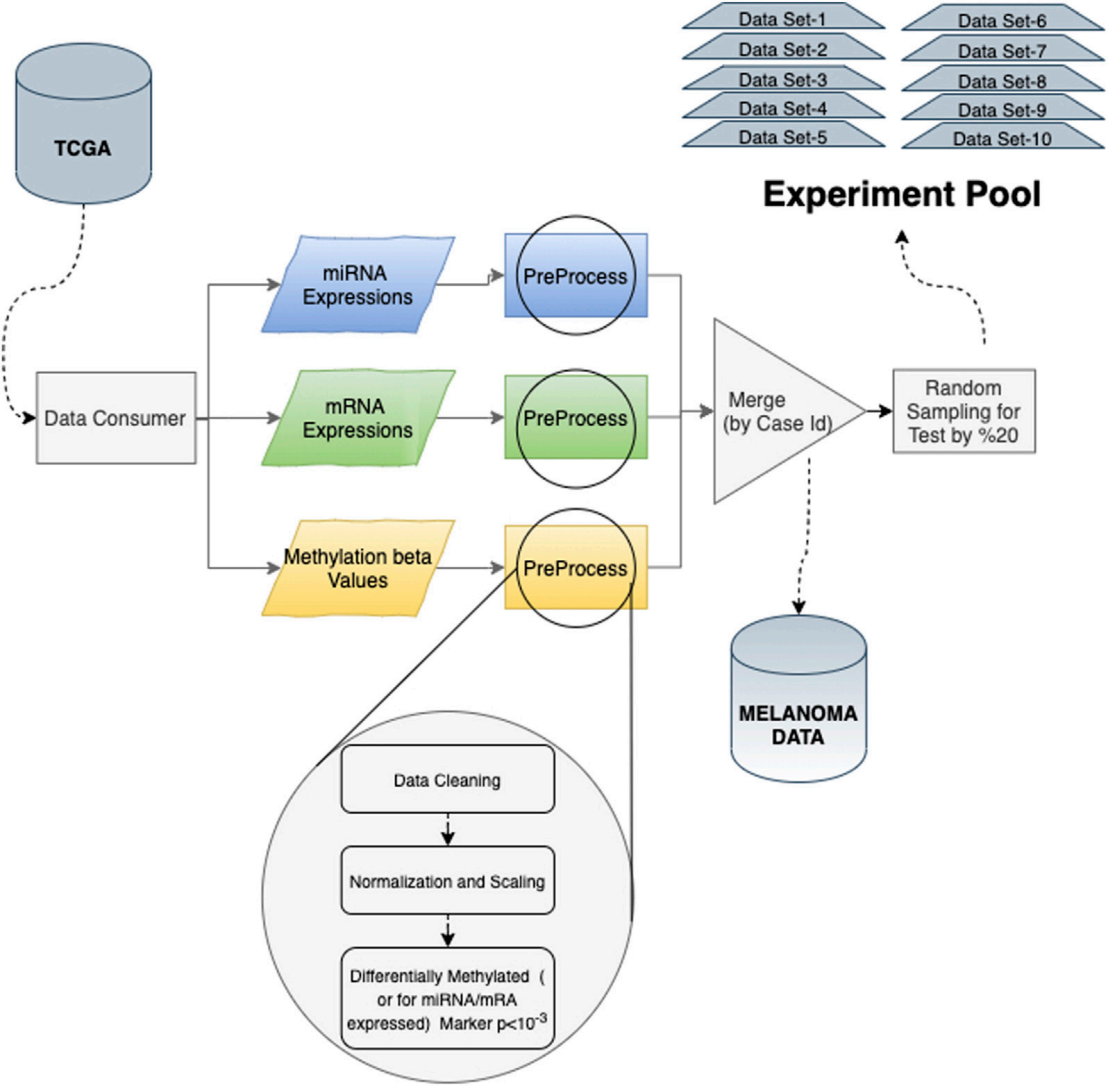
Experimental pool generation process: each method is evaluated using a sample experimental pool under the same circumstances. miRNA, mRNA, and methylation data consumed through TCGA were processed separately and merged to generate the whole melanoma marker dataset. Then, through random splinting, 10 individual sample datasets are constructed. Each random split is saved by applying both undersampling and oversampling (SMOTE) techniques.

We have collected the melanoma data for miRNA sequencing, RNA sequencing, and methylation array for this study’s systematical analysis. For 470 different cases with primary and metastatic melanoma, tissue samples are compared to distinguish the metastatic melanoma from the primary tumor. We finalized the predictive model input preprocessing by applying data cleaning, normalization, and scaling preprocessing steps for the remaining 449 cases (Figure 1). Overall, 470 distinct cases and 11,265 opened files have been found by using four filters:

1. Primary Site (Skin)
2. Project (TCGA-SKCM)
3. Experimental Strategy (miRNA-Seq; Methylation array; RNA-Seq)
4. File Access (Open)

We generated a subset of cases, which contains all data for “miRNA sequences”, “methylation array”, and “RNA sequences”. In the current interface of the GDC Data Portal, the following search query provides the data files in the repository:

Cases.primary_site in (“skin”) and cases.project.program.name in (“TCGA”) and cases.project.project_id in (“TCGA-SKCM”) and files.access in (“open”) and files.experimental_strategy in (“Methylation Array”, “RNA-Seq”, “miRNA-Seq”).

TCGA provides various attributes for “miRNA sequences”, “methylation array”, and “RNA sequences”. For miRNA, we used “miRNA Expression Quantification”, which are miRNA expressions provided as a table that associate miRNA IDs with a read count and a normalized count in reads per million miRNA mapped. Raw read counts, the number of reads aligned to each gene, calculated using the HT-Seq algorithm, are used for mRNA. Ensemble gene ID represents these data and the number of read-aligned mRNAs. For methylation analysis, TCGA provides beta-values, which approximate the percentage of methylation of the gene (Figure 1).

The data analysis is started with data preprocessing and variable selection. miRNA expression is used for the initial cycle of the spiral analysis method. Then, 11,265 separate files that contain miRNA and mRNA expressions for each case are downloaded from TCGA with a manifest file that contains metadata for the specific case. The manifest file is used to read and combine case files to generate a data pool. The final data pool contains 472 observations with 60,492 properties for mRNA, 450 observations with 1,904 properties for miRNA, and 483 observations with 34,014 variables for methylation. We only chose the cases which have all three experiments, namely, miRNA, mRNA, and methylation.

The sample type property is used for the class variable, which is a categorical variable with four levels, namely, “Primary Tumor”, “Solid Tissue Normal”, “Metastatic”, and “Additional Metastatic”. Only the samples with “Primary Tumor” and “Metastatic” are selected for further analysis.

There were variables for miRNA and mRNA expressions with a constant (1 or 0) value for all samples. These attributes have been removed from the dataset. The remaining samples are subject to a significance test concerning class variables; log normalization and Z-score normalization used for relevant markers. Markers are scaled in the 0–1 range. The *t*-test has been used as a significance test (*p*-value is defined as 0.001). As a result of the test, 425 miRNAs, 2061 mRNAs, and 8,698 methylation variables were significantly expressed between the two groups (“Primary Tumor” and “Metastatic”).

For a detailed analysis of the results, all possible miRNA patterns and their target mRNA and gene methylation are calculated. Then, depending on the significance level, different patterns are defined *via* evaluation with each other.

**FIGURE 2:**
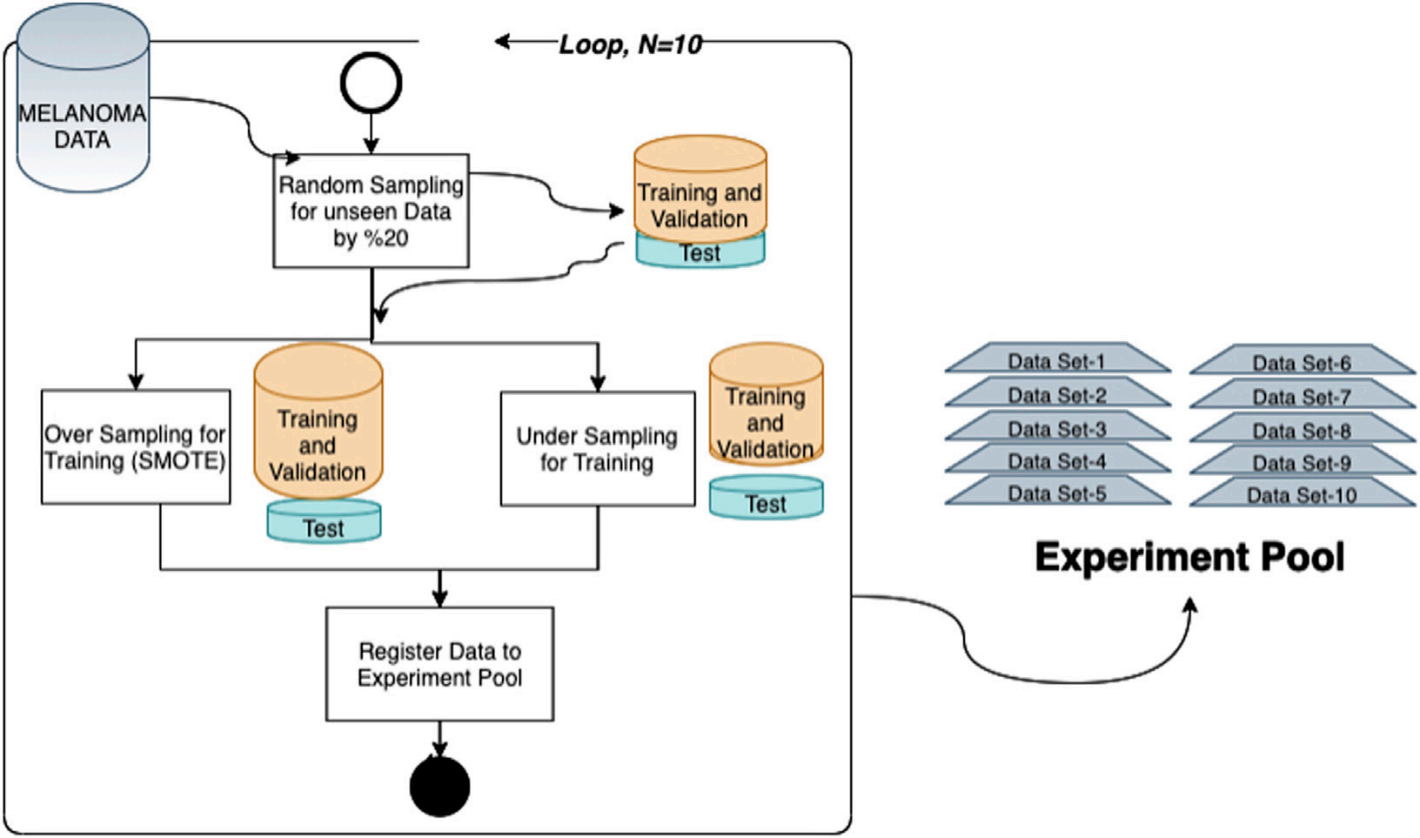
Training validation and unseen test data generation in each trial; for this purpose, the following steps are followed for both undersampling and oversampling. 1) The significant variables listed in the given category are selected from the dataset. 2) A set selected randomly from whole data with an 80% ratio of each class is kept for unseen data. 3) A technique is applied to solve the curse of the dimensionality problem (for dimensional reduction, principal component analysis is applied, and for oversampling runs, the SMOTE algorithm is used with K = 3). 4) Steps 1–3 are repeated for each data split in the experiment pool.

Random selection is applied for each class with a 20% ratio to separate unseen data for testing during the analysis. We repeated this randomization process to create 10 different splits, which are used as a separate trial. By generating more than one split, we aim to decrease the bias due to random splitting and test the repeatability. So, as an experiment environment, we created an experiment pool constructed by 10 random partitions for the test set and the training set generated by applying both undersampling and oversampling (SMOTE) (Fernández et al., 2018) techniques for addressing class imbalance issues. So, 80% of the data are used for training and validation (Figure 2). In each trial, both dimensional reduction and feature selection techniques were applied separately to solve the curse of the dimensionality problem for both undersampling and oversampling methodologies, and different machine learning techniques were evaluated with 10-fold cross-validation. Final models are tested against the unseen data separated at the beginning. All these processes were repeated 10 times for each data set in the experimental pool. Finally, the mean values of prediction parameters are calculated for the results reported in this study.

**FIGURE 3:**
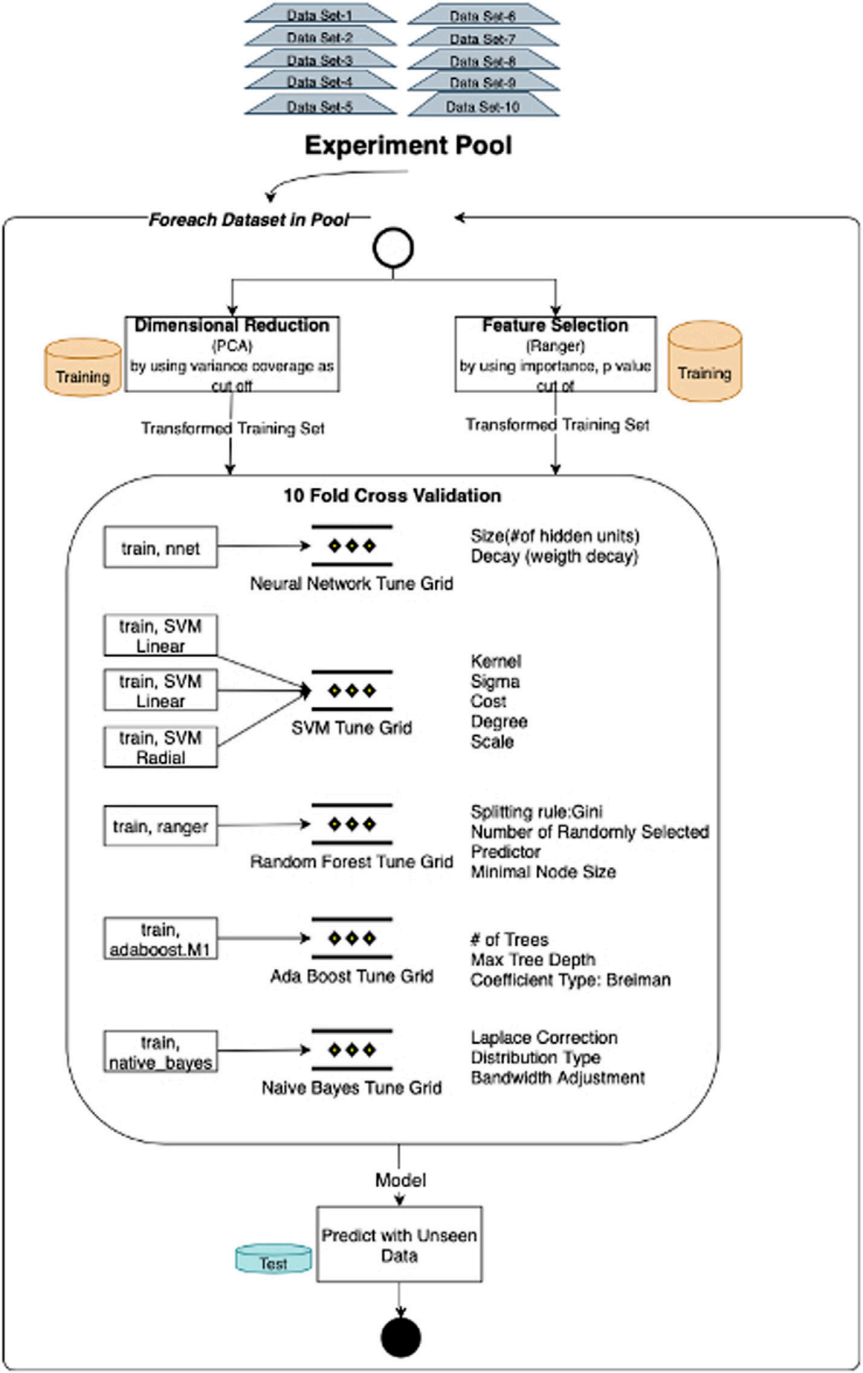
Model training and testing process: experiment flow initiated by applying alternative dimensionality solutions, namely, PCA and feature selection. Through each experiment flow, models are trained with seven (SVMs with linear, radial, and polynomial kernels, neural networks, random forests, AdaBoost, and Naive Bayes) machine learning algorithms and tested with the same unseen data. Overall flow is repeated for each data subset in the experiment pool.

Each test/training subset listed in the experiment pool was trained and tested for different models by adding miRNA expressions, mRNA expressions, and methylation beta-values iteratively. Besides, to address the curse of dimensionality, we tried both dimensional reduction and feature selection techniques. Seven methods, namely, SVMs with linear, radial, and polynomial kernels, neural networks, random forests, AdaBoost, and Naive Bayes, have been applied to generate and test the predictive model (Figure 3). Neural networks and support vector machines are frequent models that have been applied to similar classification models. But as we search the literature, we did not see any research which applied bagging, boosting, and probabilistic methods. So, we chose at least one representative of various classification algorithm categories, namely, artificial neural networks, bagging methods, boosting methods, and probabilistic models, one or more. Apart from support vector machines and neural networks, we included adaptive boosting, an ensemble method that composes a robust classifier from various weak classifiers, and random forest, which relies on bagging techniques to increase classification performance more than one decision tree. Apart from all these, Naïve Bayes also chooses an alternative since it is a fundamental model based on probabilistic techniques. The mean F-score and the mean *p*-value are evaluated as performance indicators for validation and test dataset classifications. Box plot distribution of classification scores is investigated for each dataset in the experimental pool. The best model for each category is made by comparing mean F-scores and mean *p*-values. If these results are the same for two or more best model candidates, we have reviewed the box plot of significance and sensitivity distributes.

This study follows the following coding mechanism to map the alternative scenarios of class imbalance and dimensionality solution techniques for each category. This annotation is used as the naming convention of the given result set in the following sections:

• a1: miRNA biomarkers modeled with feature selection and undersampling
• b1: miRNA biomarkers modeled with feature selection and SMOTE
• c1: miRNA biomarkers modeled with PCA and undersampling
• d1: miRNA biomarkers modeled with PCA and SMOTE
• a2: miRNA and mRNA biomarkers modeled with feature selection and undersampling
• b2: miRNA and mRNA biomarkers modeled with feature selection and SMOTE
• c2: miRNA and mRNA biomarkers modeled with PCA and undersampling
• d2: miRNA and mRNA biomarkers modeled with PCA and SMOTE
• a3: miRNA, mRNA, and methylation biomarkers modeled with feature selection and undersampling
• b3: miRNA, mRNA, and methylation biomarkers modeled with feature selection and SMOTE
• c3: miRNA, mRNA, and methylation biomarkers modeled with PCA and undersampling
• d3: miRNA, mRNA, and methylation biomarkers modeled with PCA and SMOTE

All preprocessing, training, validation, and testing are done using R studio using various R packages.

• Neural Network (package: nnet) (Ripley, 2002; Ripley and Venables, 2021)
• AdaBoost (package: adabag) (Alfaro et al., 2013; Alfaro et al., 2018)
• Random Forest (package: ranger) (Wright and Ziegler, 2017; (Wright et al., 2021)
• Naïve Bayes (package: naivebayes) (Majka and Majka, 2020)
• Support Vector Machine (package: kernlab) (Karatzoglou et al., 2004; Karatzoglou et al., 2016)
• Smote (smotefamily) (Siriseriwan, 2019a; Siriseriwan, 2019b)

**FIGURE 4:**
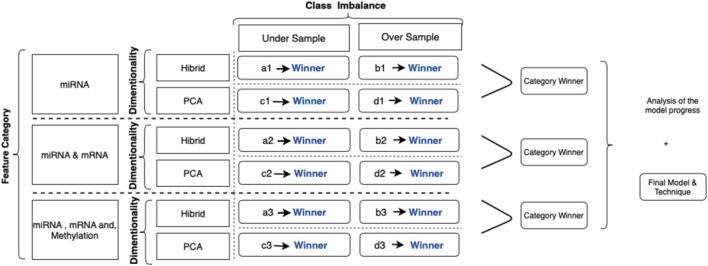
Illustration for category-based analysis with techniques applied: each evaluation criterion is represented with a code. For example, (a1) represents the predictive models by using miRNA signatures with the hybrid method, that is, the random forest, to calculate feature importance undersampling for the class imbalance solution. Similarly, (d3) represents the outcomes of models applied to predict metastasis using significant miRNA, mRNA, and methylation biomarkers using PCA as a dimensional solution and SMOTE as a class imbalance solution.

During the collection and evaluation of the results, we followed a systematic cross-comparison technique. First, we collected the prediction scores for different classification models to find the best algorithm. Evaluation of the successors within each feature category identified the winner. Finally, model progress and the contributions of adding new feature categories are assessed based on these collected results. The illustration of this process is summarized in Figure 4.

The experiment is repeated for each subset in the data pool to find the prediction scores, and mean values were calculated. We have assessed the predictive algorithm using seven different machine learning models, including representatives of various classification algorithm categories, namely, artificial neural networks, bagging methods, boosting methods, and probabilistic models. We applied 10-fold cross-validation for each subset and calculated the mean F-score; the mean *p*-value was used to evaluate each category’s best model. If the results are the same for two or more model candidates, we have reviewed the box plot of significance and sensitivity distributes to choose the one with low variance.

$$F\;Score=2*\frac{\left(Precision*Recall\right)}{Precision+Recall}$$ (1)

As a final step, we performed functional and pathway enrichment analysis by using DAVID (Huang et al., 2007; Dennis et al., 2003). The KEGG, Reactome, EC Number, and Biocarta Pathways of selected biomarkers are compared for sets of “miRNA”, “miRNA and mRNA”, and “miRNA, mRNA, and methylation” to better understand the contributing factors behind the higher precision and consistency after including methylation data in the models.

## Results

**FIGURE 5:**
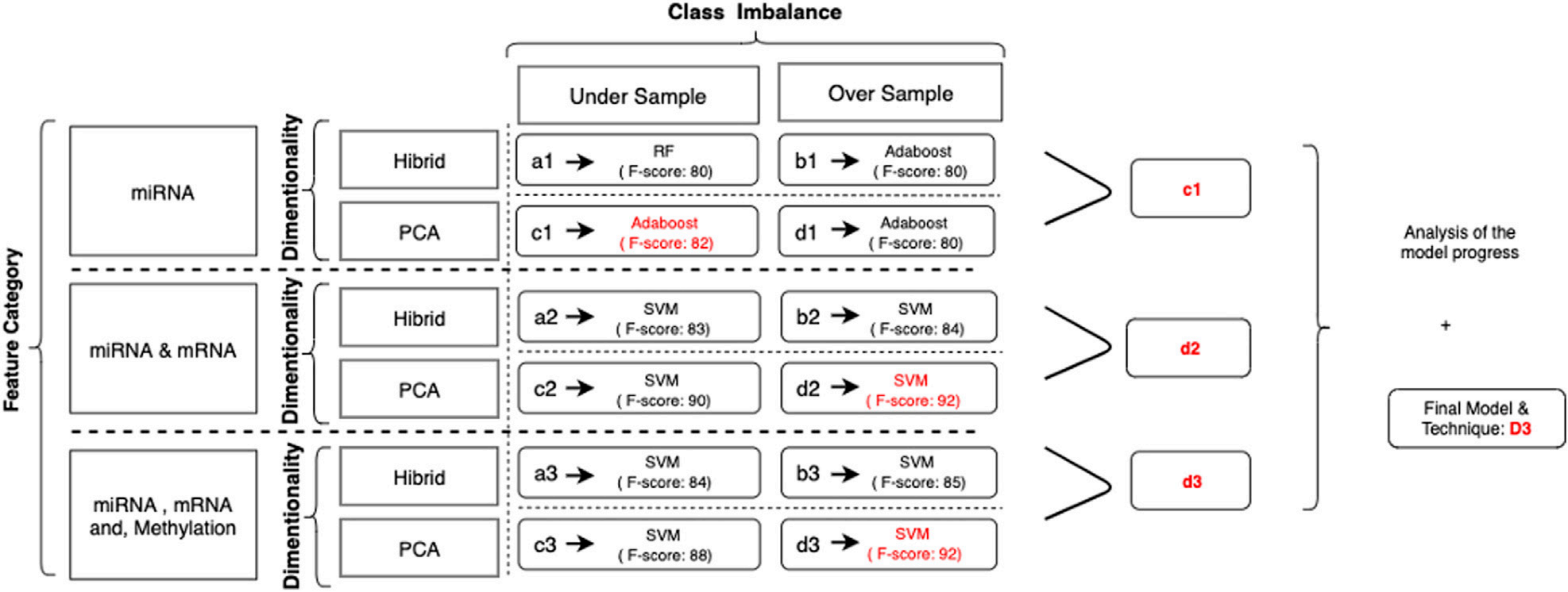
Illustration for results of category-based analysis with techniques applied to solve significant issues: as a result of the evaluation process, (c1) is selected as the successor model for miRNA markers. When two markers, miRNA and mRNA, are combined, the winner is identified as (d2). In the final cycle, the merge of all biomarkers resulted in (d3) as the successor. Among all, (d3) was the winner to predict the metastatic outcome.

In this study, we have evaluated the potential genetic biomarkers of melanoma metastasis. In addition, we developed multiple predictive models to predict the metastatic outcome by integrating miRNA, mRNA, and DNA methylation markers by using the TCGA melanoma dataset. This study’s experimental strategy is composed of a 3-cycle evaluation, each of which targets different feature categories. In each cycle, the evaluation of different techniques to solve dimensionality and the class imbalance problem is applied. Figure 5 summarizes the results of all evaluation techniques for each cycle.

**FIGURE 6:**
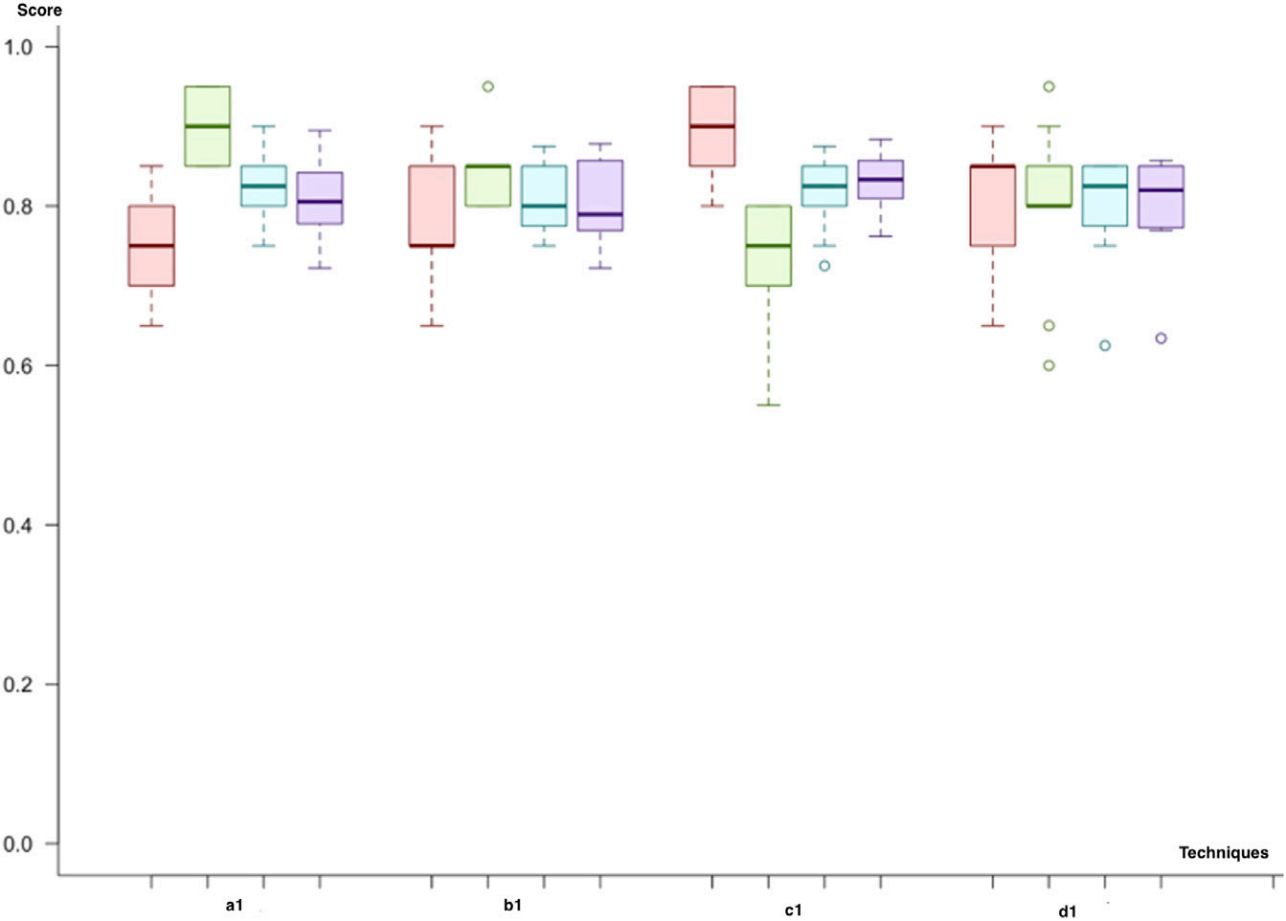
Model comparison of techniques used for miRNA biomarkers (red, sensitivity; green, predictivity; blue, accuracy; purple, F-score): Category 1, which uses a hybrid model of feature selection and an AdaBoost classifier, has the best results among all scenarios.

At the first step of the initial cycle, we have implemented a predictive model (a1) with a microRNA biomarker model using feature selection through importance (the hybrid model) and the class imbalance solution through undersampling. The predictive model with adaptive boosting (AdaBoost) demonstrates the best results among all the trials with the highest F-score and accuracy. Besides, the variance of the results for the different datasets in the experiment was also low compared to other models. Similarly, the random forest has the second-best results among all trials (an F-score of 80%). In the second scenario (b1), when we replace the class imbalance solution with SMOTE, the random forest demonstrates similar results, with an F-score of 79%. In parallel, adaptive boosting (AdaBoost) presents a comparable performance (an F-score of 80%) to that of the random forest model with a slightly higher score. In the third trial (c1), we have used undersampling and dimensional reduction with PCA. According to our results, adaptive boosting (AdaBoost) showed better scores (an F-score of 80%), but for this time, the SVM with the linear kernel (an F-score of 78%) was better than the random forest (an F-score of 72%), demonstrating the second-best results. Finally, we applied SMOTE to address the class imbalance issues (d1). The results were similar to those of the first trial; adaptive boosting showed the best results (F-score: 80%), and the random forest also had better results (F-score: 79%) than other models (Figure 6).

As a result of the initial cycle, microRNA biomarkers predict the primary tumor’s metastatic outcome with an F-score of almost 80%. In predictive models, by using miRNA markers, all workflows showed similar classification power. We selected (c1) the adaptive boosting with the PCA and undersampling as it results in the highest F-score. Both the random forest and adaptive boosting (AdaBoost) demonstrated better results in each workflow (Figure 6).

**FIGURE 7:**
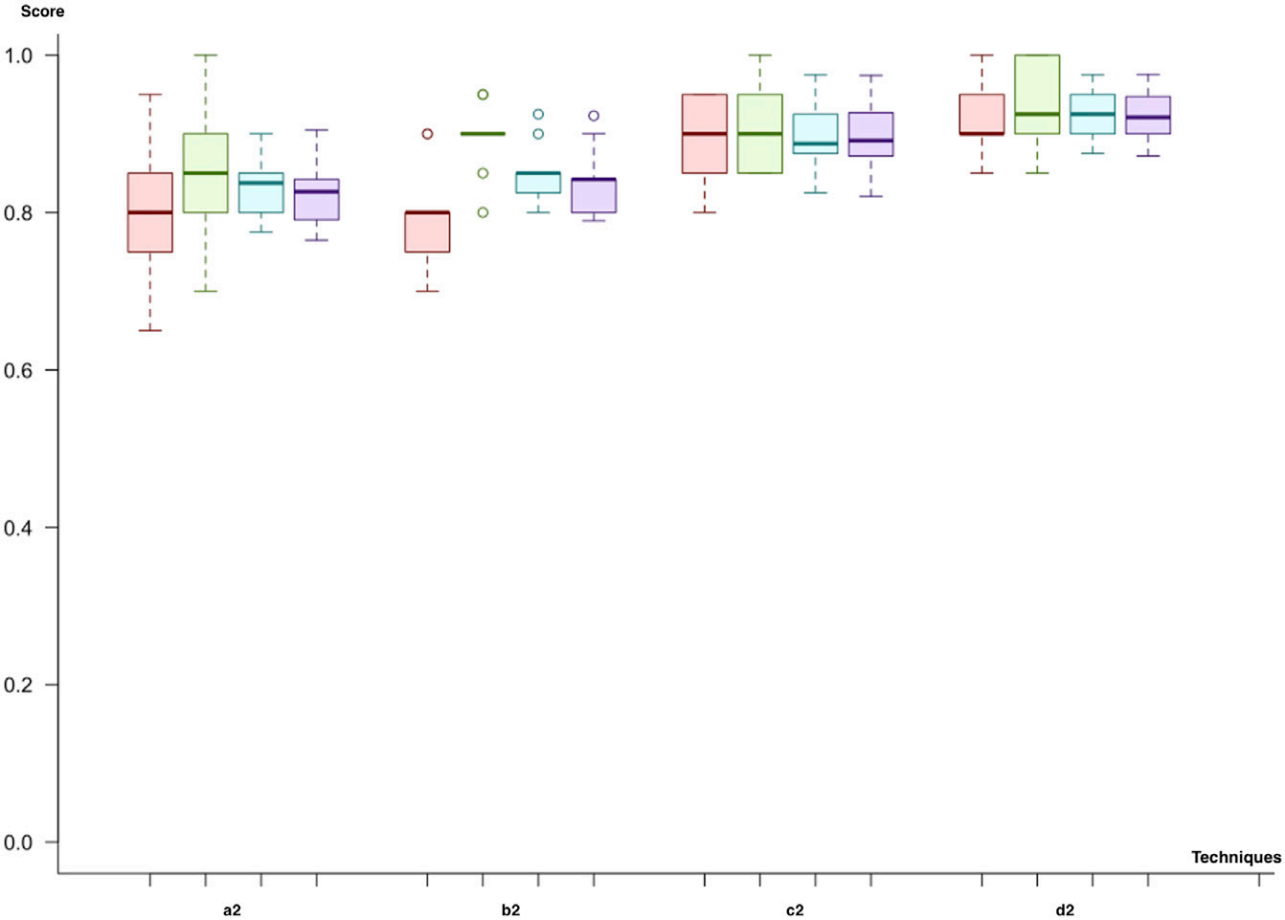
Model comparison of techniques used for miRNA and mRNA biomarkers (red, sensitivity; green, predictivity; blue, accuracy; purple, F-score): the model listed in 4, which applies (d2), is selected as the successor model for the second cycle.

In the second cycle, we utilized both miRNA and mRNA as biomarkers. Like in the previous cycle, we first used (a2) feature selection through importance (the hybrid model) and the class imbalance solution through undersampling. When we compare the predictive models, the results were quite similar, with varying F-scores between 81% and 83%. However, the random forest produces the best results with regard to the mean F-score (83%) and the mean *p*-value (8.26 × 10^−05^). The SVM with a polynomial kernel was the second-best model to predict the metastatic outcome, with the same F-score but a lower *p*-value (9.34 × 10^−05^). As a second trial (b2), we have replaced the class imbalance solution with SMOTE. The results for each model, which vary in the range of 80–84% for the F-score, were quite similar. The neural network showed the best F-score (84%) and *p*-value (2.41 × 10^−05^). The SVM with linear and polynomial kernels also had the same F-score (84%), and the neural network showed a higher significance. Adaptive boosting and the random forest demonstrate better results for the miRNA–mRNA cycle and predict the metastatic outcome with equal mean F-scores of 81%. In the third trial (c2), undersampling for class imbalance and dimensional reduction with PCA are applied. The SVM with the linear kernel was the best model with the highest F-score (90%). The neural network was the second-best model to predict metastasis with an F-score of 89%. Nevertheless, this time, adaptive boosting (F-score: 82%) and the random forest (F-score: 75%) are left behind. As the final trial (d2), we have applied SMOTE and dimensional reduction with PCA (d2). The neural network and the SVM with the linear kernel produced the best results compared to the rest with F-scores of 91 and 92%, respectively. On the other hand, adaptive boosting and the random forest showed high variance across different trials (Figure 7).

At the end of the second cycle, we saw that models using miRNA and mRNA marker winner models had F-scores ranging between 83 and 92%. The prediction scores for both boosting and bagging techniques were not as good as they were in the first cycle. Since the F-score for (d2), the SVM using PCA and SMOTE, has the highest scores, it is selected.

**FIGURE 8:**
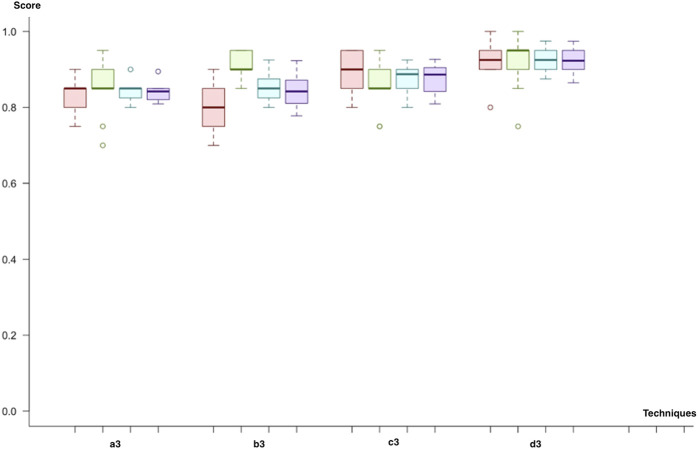
Model comparison of techniques used for miRNA, mRNA, and methylation biomarkers (red, sensitivity; green, predictivity; blue, accuracy; purple, F-score). The model listed in 4, which applies (d3), is selected as the successor model for the final cycle.

In the third cycle, we combined all miRNA, mRNA, and methylation biomarkers. Similar to previous cycles, we applied a combination of each class imbalance and dimensionality solution techniques. We decided on neural networks since model significance demonstrated improvement in our results. Firstly, all Neural network, SVM with linear and polynomial kernel predicts metastasis with an F-score of 83% by using under-sampling and feature selection through importance techniques (a3). Both SVM with radial kernel and random forest predict with similar F-scores (83%). So, the results of the prediction model were close to each other for this trial. However, the lowest variance across different trials was observed with SVM (linear kernel). In the second trial (b3), we have replaced the class imbalance solution technique with SMOTE. Both SVM with linear kernel and the polynomial kernel were the two best performing models with F-scores of 84% and 85%. In the third trial (b4), sampling and dimensional reduction with PCA are applied. SVM was the best model regardless of the selected kernel (F-score; 88%). Finally, when we applied SMOTE instead of under-sampling (d3), SVM with linear kernel demonstrated slightly higher scores (F-score 92%). In contrast, SVM with polynomial kernel and Neural network had an F-score of 91% and 90%. The best predictive model was SVM, trained by using dimensional reduction with PCA and SMOTE (d3). Like the second cycle, both SVM and Neural Network models resulted in better results in all trials. In addition, both under-sampling and oversampling techniques produced similar results (Figure 8).

**FIGURE 9:**
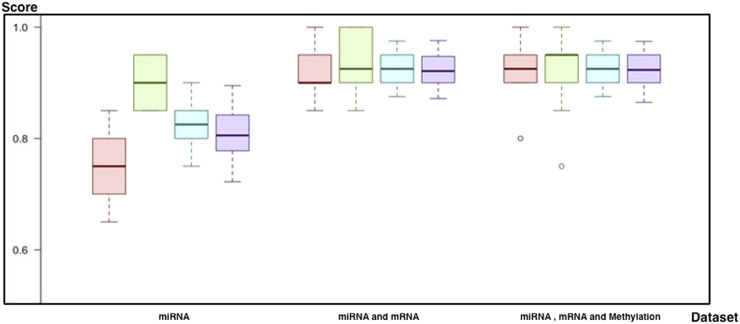
Comparison of best models for each biomarker set (red, sensitivity; green, predictivity; blue, accuracy; purple, F-score): 1) the performance of the predictive model by using miRNA, 2) the performance of the predictive model by using miRNA and mRNA markers, and 3) the performance of the predictive model by using miRNA, mRNA, and methylation markers.

As a result of all evaluations (see Supplementary Material), we came up with successors for each biomarker category (Table 1; Figure 9). First of all, the random forest with (a1) feature selection and undersampling achieved best results for miRNA markers (F-score = 81%, sensitivity = 75%, specificity = 90%, accuracy = 82%, and *p* = 1.7 × 10^−4^). In addition, the SVM (d2) with PCA and SMOTE was the most successful technique for a combination of miRNA and mRNA markers (F-score = 92%, sensitivity = 92%, specificity = 93.5%, accuracy = 93%, and *p* = 1.0 × 10^−7^). Finally, by using all miRNA, mRNA, and methylation markers (d3), the SVM reached the same results as the previous one, with higher consistency across different trials (F-score = 92%, sensitivity = 92%, specificity = 93%, accuracy = 92%, and *p* = 1.05 × 10^−7^) (Figure 9).

**TABLE 1:** Summary for iterative progress on model precision scores.

	Best method	Tuning grid	Best tune hyperparameters	Validation	Test
miRNA	PCA and undersample	Max depth: [2:8]	Max depth: 6	Accuracy: 86%	Accuracy: 81%
	AdaBoost	# of trees: [1:16]	Number of trees: 12	F-score: 86%	F-score: 82%
			Co-efficiency of learning: Breiman		
miRNA and mRNA	PCA SMOTE	Cost: 10^(−4)^ × (20:150))	Cost: 0.0025	Accuracy: 81%	Accuracy: 93%
	SVM (linear kernel)			F-score: 0.82%	F-score: 92%
miRNA, mRNA, and methylation	PCA SMOTE	Cost: 10^(−4)^ × (20:150))	Cost: 0.0027	Accuracy: 82%	Accuracy: 93%
	SVM (linear kernel)			F-score: 83%	F-score: 92%

The miRNA model applied by feature selection through importance (the hybrid model) and the class imbalance solution through undersampling is the method to be applied for prediction. For both “miRNA–mRNA” and the “miRNA–mRNA–methylation” triple model, principal component analysis for dimensionality and SMOTE for the class imbalance solution was the best method to increase predictive power and stability of the model.

In the third model, 10 miRNA biomarkers, namely, hsa-mir-142, hsa-mir-29c, hsa-mir-3124, hsa-mir-3130, hsa-mir-326, hsa-mir-331, hsa-mir-4419b, hsa-mir-4444, hsa-mir-4474, hsa-mir-4491, hsa-mir-4523, hsa-of mir-625, and hsa-mir-766, are found to be upregulated and 1 miRNA, hsa-mir-203a, was found to be downregulated. Hence, 11 miRNA markers have been used as a biomarkers in our successor model to predict metastasis. In addition, 163 methylation and 1770 mRNA markers are selected in the final triple-biomarker model. All miRNA biomarkers and their target miRNA and methylation information in their target genes are presented in Supplementary Tables S2, S3.

Evaluation of the overall results at the functional level is completed with an enrichment analysis. We used DAVID [(Huang et al., 2007; Dennis et al., 2003)] tools for biological interpretation of selected features used in the selected “miRNA and mRNA” classification and “miRNA, mRNA, and methylation” classification.

**FIGURE 10:**
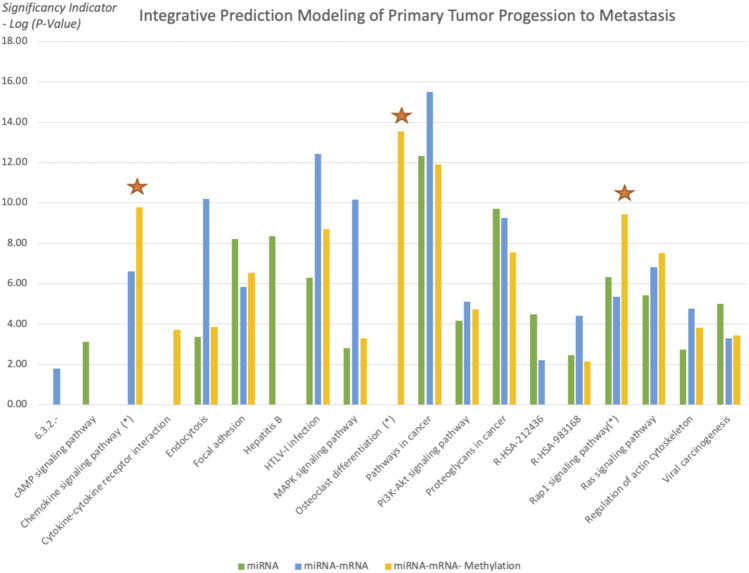
Significant pathways functionally enriched in all three feature sets. As the new biomarker set is added, the significance of the pathways is evaluated. Osteoclast, Rap1 signaling pathway, and chemokine signaling pathways showed a significant increase in the third model.

Using the functional enrichment analysis, the KEGG, Reactome, EC Number, and Biocarta Pathways of selected biomarkers are compared for “miRNA and mRNA” with “miRNA, mRNA, and methylation” to examine the reason for higher precision and consistency of addition of methylation. In the model with methylation markers, the significance of the osteoclast, Rap1 signaling pathway, and chemokine signaling pathways increased (Figure 10). Osteoclast differentiation also appealed within the top 15 pathways when all 3 biomarker categories are combined. In addition, the Rap1 signaling pathway and chemokine signaling were listed in the top 3 among the most significant pathways (Table 2).

**TABLE 2:** Comparison of the top 15 pathways of different biomarker sets.

	*p*-value		
	miRNA	miRNA–mRNA	miRNA–mRNA–methylation
6.3.2.-	—	1.60 × 10^−02^	—
cAMP signaling pathway	7.40 × 10^−04^	—	—
Chemokine signaling pathway (*)	—	2.40 × 10^−07^	1.60 10^−10^
Cytokine–cytokine receptor interaction	—	—	1.90 10^−04^
Endocytosis	4.30 × 10^−04^	6.30 × 10^−11^	1.40 10^−04^
Focal adhesion	6.10 × 10^−09^	1.40 10^−06^	2.80 10^−07^
Hepatitis B	4.40 × 10^−09^	—	—
HTLV-I infection	5.10 × 10^−07^	3.60 × 10^−13^	2.00 × 10^−09^
MAPK signaling pathway	1.60 × 10^−03^	6.70 × 10^−11^	4.90 × 10^−04^
Osteoclast differentiation (*)	—	—	2.90 × 10^−14^
Pathways in cancer	4.60 × 10^−13^	3.10 × 10^−16^	1.20 × 10^−12^
PI3K-Akt signaling pathway	7.00 × 10^−05^	8.00 × 10^−06^	1.90 × 10^−05^
Proteoglycans in cancer	2.00 × 10^−10^	5.70 × 10^−10^	2.70 × 10^−08^
R-HSA-212436	3.40 × 10^−05^	6.00 × 10^−03^	—
R-HSA-983168	3.60 × 10^−03^	3.80 × 10^−05^	7.30 × 10^−03^
Rap1 signaling pathway (*)	4.80 × 10^−07^	4.30 × 10^−06^	3.70 × 10^−10^
Ras signaling pathway	3.80 × 10^−06^	1.50 × 10^−07^	3.00 × 10^−08^
Regulation of actin cytoskeleton	1.80 × 10^−03^	1.70 × 10^−05^	1.50 × 10^−04^
Viral carcinogenesis	9.70 × 10^−06^	5.10 × 10^−04^	3.80 × 10^−04^

*p-values of the osteoclast, Rap one signaling pathway, and chemokine signaling pathways gradually increased after adding a new biomarker set. In addition, the Rap1 signaling pathway and chemokine signaling were listed among the top three pathways with increasing significance with osteoclast differentiation. Other pathways with increasing significance, such as cytokine–cytokine receptor interaction and the Ras signaling pathway, are also observed.

## Discussions

Melanoma can be distinguished with visual assessment or through a short screening. Although there is an opportunity for a cure when detected in the early stages, treatment is challenging in later stages. Likewise, metastasis is an undesired outcome in such cases, and differential diagnosis is crucial for the treatment decision. So, the opportunity for the diagnosis of metastatic melanomas in earlier stages may support therapeutic decisions and advice for more frequent and in-depth screening, providing a higher chance for cure or prevention of further metastatic progress.

This study shows that miRNA plays an essential role in the metastatic progression of primary melanoma and predicts metastasis outcomes with high accuracy. miRNA biomarkers anticipated metastatic results with an F-score of 82%. Expansion of mRNA markers upon miRNA reached an F-score of 92%. The ultimate model, which includes DNA methylation, results in a comparative F-score of 92% but delivered a steady model with low variation over different trials. Moreover, the integrated evaluation of miRNA with mRNA and methylation biomarkers increases the model’s predictive power. Another remarkable finding in this study is that the boosting and bagging model’s performance was better for miRNA signatures. However, when we added new mRNA and DNA methylation, we got higher prediction scores for neural networks and support vector machine classifiers.

One limitation of the study was the data imbalance and small sample size. We validated and tested our models in a restricted data size since we could not access additional datasets on the GEO or CGC, combining all three markers at the time of the study. We utilized oversampling techniques and ran the overall process multiple times to reduce the bias to address this limitation. Additionally, we were able to compare various machine learning models as they were appropriate for the data size in the study. However, we realize that deep learning methods would be competitive with these techniques. Therefore, repetition of the study with a balanced or more extensive dataset in the future can further validate the biomarkers reported here.

In machine learning studies, undersampling techniques are also used to deal with class imbalance issues. So we performed oversampling and undersampling methods and evaluated their outcomes. The SMOTE, a synthetic minority oversampling method based on the k-nearest neighbors, has been tested with different k values between 3 and 6, and the final k was chosen as 3. We used the 1:2 ratio for oversampling of the minority class. Under the given circumstances, we generated similar results for both undersampling and oversampling. Overall, our results present satisfactory evidence that the synthetic minority oversampling technique can also be applicable for prediction studies for genomics data.

As our model is based on the differences between primary and metastatic melanomas, the markers identified here can be used for differential diagnosis. We believe that it will become possible to predict melanomas with metastatic potential (prediction of prognosis). In those cases, several actions can be taken in the clinic, such as intensive scanning for metastasis or frequent follow-ups with patients. In the future, patients with higher risk can be offered prevention from metastasis with gene therapies based on emerging technologies like miRNA therapies or gene editing.

In this study, we focused on identifying the regulatory impact of genetic biomarkers for monitoring metastatic molecular signatures of melanoma by investigating the consolidated effect of miRNA, mRNA, and DNA methylation. We used the TCGA melanoma dataset to predict metastatic melanoma samples by assessing a set of predictive models. Differentially expressed miRNA, mRNA, and methylation signatures are used as biomarkers throughout the study. The highest performing models’ selected biomarkers are further analyzed for the biological interpretation of functional enrichment and determining regulatory networks. So we focused on gradually including new feature sets. To reveal our evaluation pattern for including new biomarker sets, we performed functional enrichment analysis. The functional enrichment of the KEGG, Reactome, EC Number, and Biocarta Pathways of selected biomarkers are compared for sets of “miRNA”, “miRNA and mRNA”, and “miRNA, mRNA, and methylation”, and we tried to search for the reason behind the higher precision and consistency achieved after addition of methylation. The osteoclast, Rap1 signaling pathway, and chemokine signaling pathways significantly increased and listed the top 15 pathways when all 3 biomarker sets are used for modeling. So the combined model populates selected biomarkers on the metastasis-associated pathways of melanoma.

Osteoclasts are multinucleated cells responsible for bone resorption. Molecular pathways involved in osteoclast proliferation, differentiation, and survival are essential players in bone metastasis. Osteoclast differentiation is a systemic pathway that controls bone renovation. Since the main metastasis sites for melanoma cancer include bone, the liver, the lung, and skin/muscle (Wright et al., 2021), functional enrichment of osteoclast-related pathways within top-level pathways is a supporting finding for our study design.

Ras-associated protein-1 (Rap1) is an important regulator for basic cell functions such as cellular migration and polarization. This pathway is an important factor for tumor metastasis, so such an increase in the significance level is also critical for the metastatic outcome (Zhang et al., 2017).

Chemokines are involved in controlling the migration of cells during normal processes of tissue maintenance or development. The chemokine-receptor system plays critical roles in various physiological processes, including immune homeostasis, inflammatory responses, and cancer progression. Chemokines have essential roles in tumor progression and are involved in the growth of many cancers and metastasis (Sarvaiya et al., 2013).

Since the initial discovery of the relationship between cancer and miRNA signatures, many studies have shown that miRNA has a critical role in the regulation of genes, and thus, has a critical role in tumorigenesis. Today, many techniques for the early detection of and diagnosis of tumors are available. Still, when invasive procedures are required for diagnosis or treatment, it is vital to know the tumor’s metastatic potential to estimate the risks vs. the benefits of the procedure. Also, in the later stages of tumor development, any information about the metastatic status of late-stage tumors is required for deciding between therapy choices. Hence, the miRNAs reported in this study can be candidates for therapeutic targets of melanoma metastasis.

## Data Availability

The datasets analyzed in this study can be found in the TCGA (The Cancer Genome Atlas Network, 2015). The names of the datasets and search methods can be found in the article. Additional information can be found in the Supplementary Material.
