# Supplementary material for: Integrative Predictive Modeling of Metastasis in Melanoma Cancer Based on MicroRNA, mRNA, and DNA Methylation Data
Source: Front Mol Biosci. 2021 Sep 23;8:637355. doi: 10.3389/fmolb.2021.637355 (PMC8495312; doi:10.3389/fmolb.2021.637355)
Supplement: Supplementary file 1 [file DataSheet1.PDF]

Supplementary Material

Table S1- Model Prediction Results For Each Experiment Cycle And Technique in Unseen Test Data

Cycle	Techniques	Model	Sensitivity	Specify	Accuracy	P Value	F Score
**miRNA**	a1	Neural Network	0.8	0.75	0.77	2.50×10^{-03}	0.77
	a1	SVM(Linear)	0.78	0.72	0.75	6.69×10^{-03}	0.75
	a1	SVM (Polynomial)	0.79	0.74	0.76	1.45×10^{-03}	0.76
	a1	SVM (Radial)	0.79	0.72	0.76	5.91×10^{-03}	0.76
	a1*	Random Forest	0.75	0.9	0.82	1.70×10^{-04}	0.81*
	a1	AdaBoost	0.78	0.79	0.78	1.59×10^{-03}	0.77
	a1	Naïve Bayes	0.56	0.89	0.73	8.77×10^{-03}	0.78
	b1	Neural Network	0.72	0.8	0.76	3.39×10^{-03}	0.75
	b1	SVM(Linear)	0.72	0.77	0.75	3.78×10^{-03}	0.74
	b1	SVM (Polynomial)	0.68	0.78	0.73	1.28×10^{-02}	0.71
	b1	SVM (Radial)	0.65	0.8	0.72	2.24×10^{-02}	0.69
	b1	Random Forest	0.71	0.92	0.81	2.59×10^{-04}	0.79
	b1*	AdaBoost	0.78	0.84	0.81	3.11×10^{-04}	0.8
	c1	Neural Network	0.68	0.79	0.73	8.79×10^{-03}	0.71
	c1	SVM(Linear)	0.8	0.74	0.77	3.97×10^{-03}	0.78
	c1	SVM (Polynomial)	0.7	0.75	0.72	1.35×10^{-02}	0.72
	c1	SVM (Radial)	0.59	0.87	0.73	1.24×10^{-02}	0.68
	c1	Random Forest	0.63	0.91	0.77	2.38×10^{-03}	0.72
	c1*	AdaBoost	0.90	0.73	0.81	4.58×10^{-04}	0.82
	d1	Neural Network	0.72	0.8	0.76	3.39×10^{-03}	0.75
	d1	SVM(Linear)	0.72	0.77	0.75	3.78×10^{-03}	0.74
	d1	SVM (Polynomial)	0.68	0.78	0.73	1.28×10^{-02}	0.71
	d1	SVM (Radial)	0.65	0.8	0.72	2.24×10^{-02}	0.69
	d1	Random Forest	0.71	0.92	0.81	2.59×10^{-04}	0.79
	d1*	AdaBoost	0.78	0.84	0.80	3.11×10^{-04}	0.80
**miRNA with mRNA**	a2	Neural Network	0.85	0.8	0.83	1.68×10^{-04}	0.83
	a2	SVM(Linear)	0.87	0.77	0.82	1.79×10^{-04}	0.83
	a2	SVM (Polynomial)	0.86	0.78	0.82	9.34×10^{-05}	0.83
	a2	SVM (Radial)	0.85	0.77	0.81	4.10×10^{-03}	0.82
	a2*	Random Forest	0.81	0.86	0.83	8.26×10^{-05}	0.83
	a2	AdaBoost	0.86	0.76	0.81	2.83×10^{-04}	0.81
	b2*	Neural Network	0.8	0.9	0.85	2.41×10^{-05}	0.84
	b2	SVM(Linear)	0.81	0.88	0.85	5.67×10^{-05}	0.84
	b2	SVM (Polynomial)	0.78	0.94	0.86	2.24×10^{-04}	0.84
	b2	SVM (Radial)	0.86	0.73	0.79	8.66×10^{-04}	0.8
	b2	Random Forest	0.72	0.93	0.82	1.70×10^{-04}	0.8
	b2	AdaBoost	0.76	0.9	0.83	3.73×10^{-04}	0.81
	c2	Neural Network	0.95	0.81	0.88	3.52×10^{-05}	0.89
	c2*	SVM(Linear)	0.9	0.91	0.9	2.40×10^{-06}	0.9
	c2	SVM (Polynomial)	0.96	0.75	0.85	1.24×10^{-04}	0.87
	c2	SVM (Radial)	0.96	0.35	0.65	2.08×10^{-01}	0.74
	c2	Random Forest	1	0.56	0.78	8.45×10^{-04}	0.82
	c2	AdaBoost	0.82	0.64	0.73	1.13×10^{-02}	0.75
**miRNA , mRNA and Methylation**	d2	Neural Network	0.93	0.88	0.9	1.40×10^{-06}	0.91
	d2*	SVM(Linear)	0.92	0.94	0.93	1.00×10^{-07}	0.92
	d2	SVM (Polynomial)	0.93	0.77	0.85	1.59×10^{-04}	0.86
	d2	SVM (Radial)	0.99	0.03	0.51	5.31×10^{-01}	0.67
	d2	Neural Network	0.96	0.6	0.78	2.50×10^{-03}	0.81
	d2	Naïve Bayes	1	0.09	0.54	3.61×10^{-01}	0.69
	d2	AdaBoost	0.8	0.74	0.77	9.23×10^{-03}	0.77
	a3	Neural Network	0.83	0.85	0.84	8.02×10^{-05}	0.84
	a3*	SVM(Linear)	0.83	0.86	0.84	2.28×10^{-05}	0.84
	a3	SVM (Polynomial)	0.87	0.81	0.84	6.41×10^{-05}	0.84
	a3	SVM (Radial)	0.84	0.82	0.83	8.86×10^{-04}	0.83
	a3	Random Forest	0.92	0.71	0.81	2.81×10^{-04}	0.83
	a3	AdaBoost	0.8	0.81	0.8	4.23×10^{-04}	0.8
	a3	Naïve Bayes	0.97	0.14	0.55	3.36×10^{-01}	0.69
	b3	Neural Network	0.77	0.92	0.84	4.51×10^{-05}	0.83

	b3	SVM(Linear)	0.78	0.93	0.85	1.51×10^{-05}	0.84
	b3*	SVM (Polynomial)	0.8	0.91	0.86	2.17×10^{-05}	0.85
	b3	SVM (Radial)	0.89	0.7	0.8	2.36×10^{-03}	0.81
	b3	Random Forest	0.75	0.76	0.75	1.62×10^{-02}	0.75
	b3	AdaBoost	0.74	0.86	0.8	2.17×10^{-03}	0.78
	b3	Naïve Bayes	0.89	0.21	0.55	3.35×10^{-01}	0.66
	c3	Neural Network	0.9	0.82	0.86	4.62×10^{-05}	0.86
	c3	SVM(Linear)	0.86	0.89	0.87	5.23×10^{-06}	0.87
	c3	SVM (Polynomial)	0.94	0.79	0.86	2.79×10^{-05}	0.87
	c3*	SVM (Radial)	0.9	0.85	0.87	1.22×10^{-05}	0.88
	c3	Random Forest	0.96	0.66	0.81	2.36×10^{-04}	0.83
	c3	AdaBoost	0.85	0.73	0.79	1.21×10^{-03}	0.79
	c3	Naïve Bayes	0.92	0.41	0.66	6.32×10^{-02}	0.73
	d3	Neural Network	0.92	0.89	0.9	2.27×10^{-06}	0.9
	d3*	SVM(Linear)	0.92	0.93	0.92	1.59×10^{-07}	0.92
	d3	SVM (Polynomial)	0.95	0.88	0.91	5.11×10^{-07}	0.91
	d3	SVM (Radial)	0.96	0.77	0.86	2.30×10^{-05}	0.88
	d3	Random Forest	0.95	0.78	0.87	3.72×10^{-06}	0.87
	d3	AdaBoost	0.85	0.85	0.85	4.01×10^{-05}	0.84

Table S2- miRNA and Methylated Gene relations presented Triple Model : *In the final model , 7 miRNA biomarkers where presented with target differentially expressed methylated genes.*

	miRNA-Methylation	***#***	***HYPER METHYLATED GENES***	***#***	***HYPO- METHYLATED GENES***
H	hsa-mir-142	3	SLC30A6, S100A11, CBD3		
	hsa-mir-3124	2	SENP1, CYP2U1		
	hsa-mir-326			1	C8orf17
	hsa-mir-331	1	IRF4		
	hsa-mir-4419b	2	TRUB2, SLC30A6		
	hsa-mir-766	5	ORC6, NDUFB5, GMPR, TMEM251, POLR1D		
L	hsa-mir-203a	1	SLC7A6OS		

Table S3- miRNA and mRNA Gene relations presented Triple Model : *In the final model , miRNA biomarkers and their target mRNA where presented together.*

	miRNA-mRNA	***#***	***upregulated mRNA***	***#***	***Downregulated mRNA***
H	hsa-mir-142	57	ENSG00000055208.16, ENSG00000065809.12, ENSG00000068366.18, ENSG00000069431.9, ENSG00000070961.13, ENSG00000084093.14, ENSG00000087460.22, ENSG00000088179.7, ENSG00000091009.7, ENSG00000096717.10, ENSG00000100934.13, ENSG00000101966.11, ENSG00000104689.8, ENSG00000105851.9, ENSG00000108064.9, ENSG00000112208.11, ENSG00000116062.13, ENSG00000116406.17, ENSG00000118058.19, ENSG00000122877.12, ENSG00000128585.16, ENSG00000128585.16, ENSG00000129450.7, ENSG00000134352.18, ENSG00000134644.14, ENSG00000134982.15, ENSG00000135913.9, ENSG00000136436.13, ENSG00000138398.14, ENSG00000140836.13, ENSG00000142512.13, ENSG00000143924.17, ENSG00000145365.10, ENSG00000147894.13, ENSG00000148516.20, ENSG00000152683.13, ENSG00000154217.13, ENSG00000158050.4, ENSG00000164164.14, ENSG00000164463.11, ENSG00000165209.17, ENSG00000165997.4, ENSG00000166211.7, ENSG00000166501.11, ENSG00000168944.14, ENSG00000171150.7, ENSG00000172493.19, ENSG00000175414.6, ENSG00000178695.5, ENSG00000179331.2, ENSG00000181450.16, ENSG00000181722.14, ENSG00000183918.13, ENSG00000185862.6, ENSG00000196233.10, ENSG00000197714.7, ENSG00000198162.11	0	-
	hsa-mir-29c	17	ENSG00000078304.18, NSG00000084093.14, ENSG00000086758.14, ENSG00000096717.10, ENSG00000117020.15, NSG00000135426.13, ENSG00000139218.16, ENSG00000143384.11, ENSG00000143970.15, ENSG00000144468.15, ENSG00000156675.14, ENSG00000158050.4, ENSG00000159873.8, ENSG00000162924.12, NSG00000164164.14, ENSG00000169756.15, ENSG00000179454.12	0	-
	hsa-mir-3124	23	ENSG00000001561.6, ENSG00000006459.9, ENSG00000028277.19, ENSG00000101347.8, ENSG00000101966.11, ENSG00000105866.12, ENSG00000111727.10 ENSG00000112486.13, ENSG00000113441.14, ENSG00000121067.16, ENSG00000134460.14, ENSG00000151789.8, ENSG00000152601.16, ENSG00000153201.14, ENSG00000154305.15, ENSG00000157827.18, ENSG00000160593.16, ENSG00000163635.16, ENSG00000164463.11, ENSG00000186265.8, ENSG00000196233.10, ENSG00000205542.9, ENSG00000273274.1	0	-
	hsa-mir-3130	12	ENSG00000089916.16, ENSG00000091009.7, ENSG00000115947.12, ENSG00000119820.9, ENSG00000130584.9, ENSG00000143751.9, ENSG00000162924.12, ENSG00000173473.9, ENSG00000174749.5, ENSG00000185155.10, ENSG00000185215.7, ENSG00000197714.7	0	-
	hsa-mir-326	10	ENSG00000009307.14, ENSG00000114331.11, ENSG00000120885.18, ENSG00000129003.14, ENSG00000143970.15, ENSG00000165997.4, ENSG00000171051.7, ENSG00000171988.16, ENSG00000172943.17 ENSG00000179813.5	0	-
	hsa-mir-331	24	ENSG00000065526.9, ENSG00000079819.15, ENSG00000085224.19, ENSG00000087460.22, ENSG00000096717.10, ENSG00000100055.19, ENSG00000103187.7, ENSG00000112658.7, ENSG00000115977.17, NSG00000116285.11, ENSG00000117500.11, ENSG00000117713.16, ENSG00000131931.7, ENSG00000136436.13, ENSG00000138443.14, ENSG00000144381.15, ENSG00000148606.11, NSG00000156976.13, NSG00000160352.14, ENSG00000162924.12, ENSG00000163960.10, ENSG00000165821.10, ENSG00000172943.17, ENSG00000241644.2	0	-
	hsa-mir-4419b	39	ENSG00000021574.10, ENSG00000074660.14, ENSG00000074706.12, ENSG00000076108.10, ENSG00000080345.16, ENSG00000081320.9, ENSG00000090659.16, ENSG00000090924.13, ENSG00000101974.13,	0	-

			NSG00000106948.15, ENSG00000108061.10, ENSG00000112658.7, ENSG00000117593.9, ENSG00000119820.9, ENSG00000120693.12, ENSG00000120868.12, ENSG00000125637.14, ENSG00000125730.15, ENSG00000131931.7, ENSG00000134070.4, ENSG00000134371.9, ENSG00000136052.8, ENSG00000136731.11, ENSG00000139146.12, ENSG00000151320.9, ENSG00000152683.13, ENSG00000158473.6, ENSG00000160049.10, ENSG00000161405.15, ENSG00000162692.9, ENSG00000163792.6, ENSG00000166822.11, ENSG00000167984.15, ENSG00000174579.3, ENSG00000178607.14, ENSG00000181896.10, ENSG00000186265.8, ENSG00000269743.2, ENSG00000273274.1		
	hsa-mir-4444	1	ENSG00000143603.17	0	-
	hsa-mir-4474	8	ENSG00000096717.10, ENSG00000116406.17, ENSG00000137478.13, ENSG00000159217.8, ENSG00000168214.19, ENSG00000168685.13, ENSG00000171150.7, ENSG00000181450.16	0	-
	hsa-mir-4491	3	ENSG00000108061.10, ENSG00000174749.5, ENSG00000181896.10	0	-
	hsa-mir-4523	1	ENSG00000198113.2	0	-
	hsa-mir-625	13	ENSG00000076108.10, ENSG00000100647.7, ENSG00000101109.10, ENSG00000102245.6, ENSG00000127124.12, ENSG00000130584.9, ENSG00000159217.8, ENSG00000159873.8, ENSG00000162885.11 , ENSG00000179454.12, ENSG00000180370.9, ENSG00000187239.15, ENSG00000254087.6	0	-
	hsa-mir-766	61	ENSG00000003436.13, ENSG00000051108.13, ENSG00000065923.8, ENSG00000074660.14, ENSG00000077044.8, ENSG00000087589.15, ENSG00000090382.5, ENSG00000090659.16, ENSG00000090924.13, ENSG00000100625.8, ENSG00000101109.10, ENSG00000101966.11, ENSG00000103522.14, ENSG00000105246.5, ENSG00000108061.10, ENSG00000108819.10, ENSG00000110395.4, ENSG00000114331.11, ENSG00000122188.11, ENSG00000124507.9, ENSG00000125107.15, ENSG00000125730.15, ENSG00000126003.6, ENSG00000126003.6, ENSG00000129450.7, ENSG00000130935.8, ENSG00000131471.5, ENSG00000132704.14, ENSG00000134070.4, ENSG00000134371.9, ENSG00000138134.10, ENSG00000138439.11, ENSG00000146112.10, ENSG00000146955.9, ENSG00000148730.6, ENSG00000150630.3, ENSG00000153147.5, ENSG00000161405.15, ENSG00000163349.20, ENSG00000164164.14, ENSG00000165821.10, ENSG00000166250.10, ENSG00000166822.11, ENSG00000167077.11, ENSG00000171408.12, ENSG00000175414.6, ENSG00000176390.11, NSG00000178385.12, ENSG00000178502.5, ENSG00000180616.7, ENSG00000181450.16, ENSG00000181896.10, ENSG00000183484.10, ENSG00000186265.8, ENSG00000188994.11, ENSG00000197057.7, ENSG00000197714.7, ENSG00000239264.7, ENSG00000243811.6, ENSG00000273274.1, ENSG00000278195.1	0	-
L	hsa-mir-203a	40	ENSG00000004468.11, ENSG00000049249.7, ENSG00000068796.15, ENSG00000087460.22, ENSG00000100985.7, ENSG00000104432.11, ENSG00000106483.10, ENSG00000108854.14, ENSG00000116473.13, ENSG00000116473.13, ENSG00000117713.16, ENSG00000118260.13, ENSG00000119004.13, ENSG00000120693.12, ENSG00000121879.3, ENSG00000125630.14, ENSG00000126003.6, ENSG00000126778.8, ENSG00000128512.18, ENSG00000139146.12, ENSG00000142875.18, ENSG00000143190.20, ENSG00000148516.20, ENSG00000152601.16, ENSG00000153561.11, ENSG00000157827.18, ENSG00000158985.12, ENSG00000160791.13, ENSG00000163848.17, ENSG00000173166.16, ENSG00000175471.18, ENSG00000179454.12, ENSG00000185551.11, ENSG00000187800.12, ENSG00000188452.12, ENSG00000196233.10, ENSG00000196428.11, ENSG00000196428.11, ENSG00000205268.9 , ENSG00000266412.4	0	-

Table S4- Differentially Methylated Genes Selected in Triple Model: In the final model there was 70 Hypermethylated genes and 75 Hypomethylated genes that does not target any miRNA

	#	
Hyper Methylated Genes	70	AC004381.6, FAM214B, HMG20B, RABL2B, P2RX7, MCAT, RP1-191J18.66, UBXN8, VPS4B, CAAP1, ZNFX1-AS1_2, USP8, AP1AR, TPRKB, SLC30A5, ZC3H12C, Z98744.1, UEVLD, AAED1, BAP1, UBAP1, FAM161A, ETFDH, GOLGB1, CTD-2026K11.4, ZNF223, PTTG1IP, RPL23AP82, PIGP, AMZ2, FAM179B, SNORD1B, RNU6-560P, SNORD14B, HIST2H2BC, MIR636, MIR124-3, MIR3074, SNORD12C, ZNF845, AC079305.10, AC105247.1, NSUN5P1, Z93930.1, PET100, CTD-2192J16.11, CTB-187M2.1, AC013264.2,

		NDUFS5P2, RPL29P30, ARHGEF38, SMARCA5-AS1, CRNDE, ZNF564, AC004066.3, AC083843.2, AF186192.1, AF186192.5, CYP4A44P, LINC01003, SNORD3B-2, MIR3655, MIR4795, AC005625.1, CTB-129P6.11, CTD-2371O3.3, AC003956.1, CTC-339O9.2, CTD-2562G15.3, MIR6892
Hypo Methylated Genes	75	CTD-2555A7.3, SPO11, ZP2, SLAMF1, TTF1, HRH4, FBXO10, MS4A1, CCL15-CCL14, CRYGC, MS4A5, CCL23, C7orf33, OR5A2, ANKS4B, RPL24P4, KRTAP6-1, PRG2, KRTAP22-1, KRTAP6-2, KRTAP19-4, KRTAP15-1, KRTAP21-1, MPEG1, DCLRE1A, RNU6-940P, RN7SKP249, GCNT6, KRTAP5-6, KRTAP20-3, KRTAP20-4, KRTAP271, TRAJ43, TRAJ42, TRAJ29, TRAJ24, AC005229.7, NUTF2P5, AP000357.4, ARL5AP4, SUCLA2P1, AC004899.3, IGBP1P1, THRAP3P1, AC013436.6, LINC00692, RP11-101C11.1, AC113610.1, SPA17P1, KLF7-IT1, RN7SL337P, ERVFRD-1, KRTAP20-1, TRAJ60, BRD9P2, CTD-2083E4.5, RTEL1P1, RNU6ATAC32P, RNU6-327P, SERPINE3, KB-1107E3.1, KB-1615E4.2, CTC-535M15.2, LINC00520, CTD-2033D15.1, HLA-P, AF001550.7, MIR4288, RN7SL612P, CCL15, GS1-21A4.2, MIR7848, CTD-2373N4.3, FLJ42393, LINC01584

Table S5- Differentially Expressed miRNA Selected in Triple Model: In the final model there was 1529 upregulated genes and 9 downregulated mRNA which is not target of any miRNA

	#	
Down regulated mRNA	9	ENSG00000137675.4, ENSG00000164687.9, ENSG00000168703.5, ENSG00000178184.14, ENSG00000180921.6, ENSG00000198695.2, ENSG00000226145.6, ENSG00000262133.1, ENSG00000276023.3,
Up regulated RNA	1529	ENSG00000000938.11, ENSG00000002933.6, ENSG00000003096.12, ENSG00000003400.13, ENSG00000003402.18, ENSG00000005020.11, ENSG00000005700.13, ENSG00000005844.16, ENSG00000007129.16, ENSG00000007312.11, ENSG00000007314.10, ENSG00000008277.13, ENSG00000008952.15, ENSG00000009790.13, ENSG00000010610.8, ENSG00000010671.14, ENSG00000010818.7, ENSG00000011590.12, ENSG00000011600.10, ENSG00000012779.9, ENSG00000013725.13, ENSG00000015133.17, ENSG00000015285.9, ENSG00000019169.10, ENSG00000022267.15, ENSG00000023902.12, ENSG00000024048.9, ENSG00000025434.17, ENSG00000026103.18, ENSG00000026297.14, ENSG00000026751.15, ENSG00000027075.12, ENSG00000028137.15, ENSG00000030066.12, ENSG00000030419.15, ENSG00000032219.17, ENSG00000033170.15, ENSG00000033178.11, ENSG00000033800.12, ENSG00000035720.6, ENSG00000037749.10, ENSG00000038427.14, ENSG00000038945.13, ENSG00000039123.14, ENSG00000039537.12, ENSG00000040199.17, ENSG00000040933.14, ENSG00000042980.11, ENSG00000043462.10, ENSG00000047410.12, ENSG00000047457.12, ENSG00000048462.9, ENSG00000048991.15, ENSG00000049768.13, ENSG00000054219.10, ENSG00000054267.19, ENSG00000054282.14, ENSG00000055163.17, ENSG00000055917.14, ENSG00000056097.14, ENSG00000056277.14, ENSG00000057657.13, ENSG00000058063.14, ENSG00000058091.15, ENSG00000058272.14, ENSG00000060982.13, ENSG00000061918.11, ENSG00000062650.16, ENSG00000064218.4, ENSG00000064989.11, ENSG00000065328.15, ENSG00000065534.17, ENSG00000065613.12, ENSG00000065675.13, ENSG00000065717.13, ENSG00000065882.14, ENSG00000066056.12, ENSG00000066294.13, ENSG00000066336.10, ENSG00000066422.4, ENSG00000066583.10, ENSG00000067369.12, ENSG00000068784.11, ENSG00000068831.17, ENSG00000068976.12, ENSG00000069122.17, ENSG00000069275.12, ENSG00000070190.11, ENSG00000070915.8, ENSG00000071054.14, ENSG00000071073.11, ENSG00000071246.9, ENSG00000072401.13, ENSG00000072736.17, ENSG00000072818.10, ENSG00000072858.9, ENSG00000073614.10, ENSG00000073754.5, ENSG00000073849.13, ENSG00000073861.2, ENSG00000074370.16, ENSG00000074966.9, ENSG00000075151.18, ENSG00000075213.9, ENSG00000075420.11, ENSG00000076662.8, ENSG00000076770.13, ENSG00000077097.12, ENSG00000077420.14, ENSG00000078177.12, ENSG00000078269.12, ENSG00000078589.11, ENSG00000078674.16, ENSG00000079263.17, ENSG00000079335.16, ENSG00000080200.8, ENSG00000080298.14, ENSG00000081019.12, ENSG00000081189.12, ENSG00000081237.17, ENSG00000082074.14, ENSG00000083168.8, ENSG00000083454.20, ENSG00000083799.16, ENSG00000083828.14, ENSG00000084070.10, ENSG00000084676.14, ENSG00000085265.9, ENSG00000085276.16, ENSG00000085514.14, ENSG00000086200.15, ENSG00000086730.15, ENSG00000087303.15, ENSG00000088205.11, ENSG00000088340.14, ENSG00000088827.11, ENSG00000089012.13, ENSG00000089505.16, ENSG00000089639.9, ENSG00000089820.14, ENSG00000090060.16, ENSG00000091106.17, ENSG00000091317.7, ENSG00000091490.9, ENSG00000092051.15, ENSG00000092871.15, ENSG00000093072.14, ENSG00000093217.8, ENSG00000095002.11, ENSG00000095370.18, ENSG00000095574.10, ENSG00000095951.15, ENSG00000096654.14, ENSG00000099250.16, ENSG00000099308.9, ENSG00000099715.13, ENSG00000100060.16, ENSG00000100079.6, ENSG00000100122.5, ENSG00000100234.11, ENSG00000100281.12, ENSG00000100336.16, ENSG00000100346.16, ENSG00000100365.13, ENSG00000100368.12, ENSG00000100385.12, ENSG00000100578.13, ENSG00000100580.7, ENSG00000100600.13, ENSG00000100628.10, ENSG00000100629.15, ENSG00000100731.14, ENSG00000100815.11, ENSG00000101017.12, ENSG00000101082.12, ENSG00000101307.14, ENSG00000101310.13, ENSG00000101336.11, ENSG00000101916.11, ENSG00000101972.17, ENSG00000102043.14, ENSG00000102096.9, ENSG00000102401.18, ENSG00000102524.10, ENSG00000102755.9, ENSG00000102879.14, ENSG00000102893.14, ENSG00000103365.14, ENSG00000103479.13, ENSG00000103540.15, ENSG00000103657.12, ENSG00000104043.13, ENSG00000104133.13, ENSG00000104213.11, ENSG00000104814.11, ENSG00000104894.10, ENSG00000104903.4, ENSG00000104972.13, ENSG00000104974.9, ENSG00000105122.11, ENSG00000105329.8, ENSG00000105339.9, ENSG00000105366.14, ENSG00000105369.8, ENSG00000105501.10, ENSG00000105639.17, ENSG00000105738.9, ENSG00000105967.14, ENSG00000106415.11, ENSG00000106511.5, ENSG00000106537.7, ENSG00000106560.9, ENSG00000106565.16, ENSG00000106952.6, ENSG00000107099.14, ENSG00000107290.12, ENSG00000107562.15, ENSG00000107581.11, ENSG00000107625.11, ENSG00000107669.16, ENSG00000107736.18, ENSG00000107742.11, ENSG00000107798.16, ENSG00000107864.13, ENSG00000107890.15, ENSG00000108055.9, ENSG00000108370.14, ENSG00000108405.3, ENSG00000108506.10, ENSG00000108510.8, ENSG00000108622.9, ENSG00000108798.7, ENSG00000108946.13, ENSG00000109320.10, ENSG00000109436.7, ENSG00000109684.13, ENSG00000109943.7, ENSG00000110077.13, ENSG00000110079.15, ENSG00000110324.8, ENSG00000110422.10, ENSG00000110448.9, ENSG00000110777.10, ENSG00000110799.12, ENSG00000110848.7,

ENSG00000110876.9, ENSG00000110934.9, ENSG00000111144.8, ENSG00000111262.4, ENSG00000111348.7, ENSG00000111644.6, ENSG00000111647.11, ENSG00000111679.15, ENSG00000111729.11, ENSG00000111796.3, ENSG00000111817.15, ENSG00000111879.17, ENSG00000111885.6, ENSG00000111912.17, ENSG00000112195.8, ENSG00000112214.9, ENSG00000112303.12, ENSG00000112406.4, ENSG00000112624.11, ENSG00000112782.14, ENSG00000112799.7, ENSG00000112936.17, ENSG00000112964.12, ENSG00000113088.5, ENSG00000113263.11, ENSG00000113269.12, ENSG00000113368.10, ENSG00000113532.11, ENSG00000113555.5, ENSG00000113595.13, ENSG00000113810.14, ENSG00000114013.14, ENSG00000114127.9, ENSG00000114439.17, ENSG00000114850.5, ENSG00000114978.16, ENSG00000115020.15, ENSG00000115085.12, ENSG00000115159.14, ENSG00000115165.8, ENSG00000115232.12, ENSG00000115271.9, ENSG00000115355.14, ENSG00000115421.11, ENSG00000115464.13, ENSG00000115604.9, ENSG00000115607.8, ENSG00000115760.12, ENSG00000115816.12, ENSG00000115935.15, ENSG00000115956.9, ENSG00000115966.15, ENSG00000115970.17, ENSG00000116017.9, ENSG00000116127.16, ENSG00000116584.16, ENSG00000116678.17, ENSG00000116701.13, ENSG00000116747.11, ENSG00000116748.18, ENSG00000116824.4, ENSG00000116852.13, ENSG00000116984.11, ENSG00000117000.8, ENSG00000117091.8, ENSG00000117114.18, ENSG00000117115.11, ENSG00000117215.13, ENSG00000117335.17, ENSG00000117523.14, ENSG00000117594.8, ENSG00000117697.13, ENSG00000118007.11, ENSG00000118292.7, ENSG00000118308.13, ENSG00000118407.13, ENSG00000118412.11, ENSG00000118495.17, ENSG00000118816.8, ENSG00000118849.8, ENSG00000118873.14, ENSG00000118922.15, ENSG00000119285.9, ENSG00000119397.15, ENSG00000119535.16, ENSG00000119699.6, ENSG00000119778.13, ENSG00000119844.13, ENSG00000119927.12, ENSG00000120063.8, ENSG00000120071.11, ENSG00000120262.9, ENSG00000120279.6, ENSG00000120280.5, ENSG00000120436.3, ENSG00000120519.13, ENSG00000120659.13, ENSG00000120802.12, ENSG00000120899.16, ENSG00000120907.16, ENSG00000121104.6, ENSG00000121210.14, ENSG00000121281.11, ENSG00000121361.3, ENSG00000121380.11, ENSG00000121481.9, ENSG00000121486.10, ENSG00000121577.12, ENSG00000121594.10, ENSG00000121797.9, ENSG00000121807.5, ENSG00000121895.7, ENSG00000121966.6, ENSG00000121988.16, ENSG00000122008.14, ENSG00000122025.13, ENSG00000122122.9, ENSG00000122223.11, ENSG00000122224.16, ENSG00000122482.19, ENSG00000122862.4, ENSG00000122986.12, ENSG00000123066.6, ENSG00000123329.16, ENSG00000123338.11, ENSG00000123411.13, ENSG00000123607.13, ENSG00000123636.16, ENSG00000124019.9, ENSG00000124191.16, ENSG00000124196.5, ENSG00000124203.5, ENSG00000124256.13, ENSG00000124496.11, ENSG00000124789.10, ENSG00000125245.11, ENSG00000125354.21, ENSG00000125384.6, ENSG00000125686.10, ENSG00000125735.9, ENSG00000125810.9, ENSG00000125900.11, ENSG00000125910.5, ENSG00000126264.8, ENSG00000126353.3, ENSG00000126759.11, ENSG00000126777.16, ENSG00000126860.10, ENSG00000126970.14, ENSG00000127084.16, ENSG00000127311.8, ENSG00000128218.7, ENSG00000128262.7, ENSG00000128271.18, ENSG00000128313.2, ENSG00000128340.13, ENSG00000128604.17, ENSG00000128815.16, ENSG00000128917.6, ENSG00000128923.9, ENSG00000129173.11, ENSG00000129515.17, ENSG00000129534.12, ENSG00000129675.14, ENSG00000130024.13, ENSG00000130038.8, ENSG00000130224.13, ENSG00000130592.12, ENSG00000130755.11, ENSG00000130830.13, ENSG00000131042.12, ENSG00000131378.12, ENSG00000131401.10, ENSG00000131725.12, ENSG00000131979.17, ENSG00000132334.15, ENSG00000132465.9, ENSG00000132514.12, ENSG00000132965.8, ENSG00000133116.7, ENSG00000133216.15, ENSG00000133246.10, ENSG00000133302.11, ENSG00000133422.11, ENSG00000133561.14, ENSG00000133574.8, ENSG00000133878.7, ENSG00000134061.5, ENSG00000134072.9, ENSG00000134109.9, ENSG00000134242.14, ENSG00000134470.18, ENSG00000134516.14, ENSG00000134539.15, ENSG00000134602.14, ENSG00000134744.12, ENSG00000134987.10, ENSG00000135077.7, ENSG00000135297.14, ENSG00000135338.12, ENSG00000135362.12, ENSG00000135439.10, ENSG00000135636.12, ENSG00000135829.15, ENSG00000135837.14, ENSG00000135932.9, ENSG00000135968.18, ENSG00000135999.10, ENSG00000136167.12, ENSG00000136237.17, ENSG00000136250.10, ENSG00000136286.13, ENSG00000136367.13, ENSG00000136404.14, ENSG00000136560.12, ENSG00000136603.12, ENSG00000136628.16, ENSG00000136634.5, ENSG00000136709.10, ENSG00000136840.17, ENSG00000136869.13, ENSG00000137078.7, ENSG00000137101.11, ENSG00000137414.5, ENSG00000137462.6, ENSG00000137491.13, ENSG00000137757.9, ENSG00000137841.10, ENSG00000137868.17, ENSG00000137962.11, ENSG00000138061.10, ENSG00000138071.12, ENSG00000138078.14, ENSG00000138160.5, ENSG00000138182.13, ENSG00000138190.15, ENSG00000138336.8, ENSG00000138378.16, ENSG00000138411.9, ENSG00000138449.9, ENSG00000138615.5, ENSG00000138684.6, ENSG00000138688.14, ENSG00000138735.14, ENSG00000138755.5, ENSG00000138767.11, ENSG00000138778.10, ENSG00000138792.8, ENSG00000138814.15, ENSG00000138964.15, ENSG00000139182.12, ENSG00000139187.8, ENSG00000139193.3, ENSG00000139194.6, ENSG00000139278.8, ENSG00000139436.19, ENSG00000139567.11, ENSG00000139610.1, ENSG00000139626.14, ENSG00000139725.6, ENSG00000139910.18, ENSG00000139985.6, ENSG00000140030.5, ENSG00000140199.10, ENSG00000140285.8, ENSG00000140368.11, ENSG00000140396.11, ENSG00000140548.8, ENSG00000140678.15, ENSG00000140749.8, ENSG00000140835.9, ENSG00000140968.9, ENSG00000141068.12, ENSG00000141161.10, ENSG00000141293.14, ENSG00000141480.16, ENSG00000141506.12, ENSG00000141968.6, ENSG00000142347.15, ENSG00000143110.10, ENSG00000143119.11, ENSG00000143156.12, ENSG00000143157.10, ENSG00000143162.7, ENSG00000143167.10, ENSG00000143185.3, ENSG00000143195.11, ENSG00000143226.12, ENSG00000143297.17, ENSG00000143344.14, ENSG00000143498.16, ENSG00000143815.13, ENSG00000143851.14, ENSG00000144028.13, ENSG00000144130.10, ENSG00000144228.7, ENSG00000144426.17, ENSG00000144476.5, ENSG00000144711.12, ENSG00000144909.7, ENSG00000145041.14, ENSG00000145088.7, ENSG00000145246.12, ENSG00000145287.9, ENSG00000145332.12, ENSG00000145416.12, ENSG00000145649.7, ENSG00000145703.14, ENSG00000145715.13, ENSG00000145730.19, ENSG00000145734.17, ENSG00000145779.7, ENSG00000145850.7, ENSG00000146070.15, ENSG00000146094.12, ENSG00000146192.13, ENSG00000146247.13, ENSG00000146476.9, ENSG00000146966.11, ENSG00000147010.16, ENSG00000147124.11, ENSG00000147138.1, ENSG00000147168.11, ENSG00000147251.14, ENSG00000147443.11, ENSG00000147570.8, ENSG00000148700.12, ENSG00000148835.10, ENSG00000148948.6, ENSG00000149781.11, ENSG00000150337.12, ENSG00000150636.14, ENSG00000150637.7, ENSG00000150681.8, ENSG00000150961.13, ENSG00000150977.10, ENSG00000150995.16, ENSG00000151461.18, ENSG00000151490.12, ENSG00000151612.14, ENSG00000151702.15, ENSG00000151779.11, ENSG00000151835.12, ENSG00000152061.20, ENSG00000152213.3, ENSG00000152315.4, ENSG00000152404.14, ENSG00000152495.9, ENSG00000152804.9, ENSG00000152969.15, ENSG00000153012.10, ENSG00000153015.14, ENSG00000153107.10, ENSG00000153214.8, ENSG00000153234.12,

ENSG00000153283.11, ENSG00000153551.12, ENSG00000153563.14, ENSG00000153574.8, ENSG00000153827.12, ENSG00000154001.12, ENSG00000154016.12, ENSG00000154065.15, ENSG00000154122.11, ENSG00000154451.13, ENSG00000154736.5, ENSG00000154822.14, ENSG00000154978.11, ENSG00000155304.5, ENSG00000155307.16, ENSG00000155330.8, ENSG00000155465.17, ENSG00000155629.13, ENSG00000155640.6, ENSG00000155659.13, ENSG00000155849.14, ENSG00000155926.12, ENSG00000155962.11, ENSG00000156136.8, ENSG00000156218.11, ENSG00000156531.15, ENSG00000156650.11, ENSG00000156869.11, ENSG00000156876.9, ENSG00000157107.12, ENSG00000157303.9, ENSG00000157450.14, ENSG00000157554.17, ENSG00000158270.11, ENSG00000158517.12, ENSG00000158714.9, ENSG00000158717.9, ENSG00000158850.13, ENSG00000159189.10, ENSG00000159314.10, ENSG00000159459.10, ENSG00000159618.14, ENSG00000159640.13, ENSG00000159753.12, ENSG00000159904.10, ENSG00000160185.12, ENSG00000160219.10, ENSG00000160224.15, ENSG00000160255.15, ENSG00000160654.8, ENSG00000160856.19, ENSG00000160883.9, ENSG00000161640.14, ENSG00000161929.13, ENSG00000161940.9, ENSG00000162434.10, ENSG00000162511.7, ENSG00000162614.17, ENSG00000162618.11, ENSG00000162654.8, ENSG00000162687.15, ENSG00000162711.15, ENSG00000162739.12, ENSG00000162775.13, ENSG00000162999.11, ENSG00000163029.14, ENSG00000163110.13, ENSG00000163125.14, ENSG00000163131.9, ENSG00000163154.5, ENSG00000163214.19, ENSG00000163219.10, ENSG00000163249.8, ENSG00000163297.15, ENSG00000163322.12, ENSG00000163376.10, ENSG00000163510.12, ENSG00000163518.9, ENSG00000163519.12, ENSG00000163563.7, ENSG00000163564.13, ENSG00000163600.11, ENSG00000163606.9, ENSG00000163611.10, ENSG00000163625.14, ENSG00000163638.12, ENSG00000163823.3, ENSG00000163947.10, ENSG00000164056.9, ENSG00000164088.16, ENSG00000164116.15, ENSG00000164134.11, ENSG00000164167.8, ENSG00000164330.15, ENSG00000164430.14, ENSG00000164483.15, ENSG00000164691.15, ENSG00000164749.10, ENSG00000165140.8, ENSG00000165168.7, ENSG00000165178.9, ENSG00000165288.10, ENSG00000165406.14, ENSG00000165457.12, ENSG00000165632.7, ENSG00000165685.7, ENSG00000165694.8, ENSG00000165813.15, ENSG00000166002.5, ENSG00000166086.11, ENSG00000166128.11, ENSG00000166263.12, ENSG00000166341.7, ENSG00000166439.5, ENSG00000166448.13, ENSG00000166478.8, ENSG00000166734.17, ENSG00000166927.11, ENSG00000166963.11, ENSG00000167083.5, ENSG00000167208.13, ENSG00000167261.12, ENSG00000167286.8, ENSG00000167613.14, ENSG00000167618.8, ENSG00000167635.10, ENSG00000167664.7, ENSG00000167850.3, ENSG00000167851.12, ENSG00000167861.14, ENSG00000167874.6, ENSG00000167895.13, ENSG00000168016.12, ENSG00000168071.20, ENSG00000168081.7, ENSG00000168229.3, ENSG00000168310.9, ENSG00000168386.17, ENSG00000168404.11, ENSG00000168405.13, ENSG00000168421.11, ENSG00000168438.13, ENSG00000168497.4, ENSG00000168813.15, ENSG00000168918.12, ENSG00000168995.12, ENSG00000169403.10, ENSG00000169413.2, ENSG00000169442.7, ENSG00000169508.6, ENSG00000169896.15, ENSG00000170458.12, ENSG00000170476.14, ENSG00000170485.15, ENSG00000170525.17, ENSG00000170571.10, ENSG00000170776.18, ENSG00000170909.12, ENSG00000170989.8, ENSG00000171049.8, ENSG00000171105.12, ENSG00000171115.3, ENSG00000171227.6, ENSG00000171522.5, ENSG00000171631.13, ENSG00000171643.12, ENSG00000171657.5, ENSG00000171659.12, ENSG00000171777.14, ENSG00000171860.4, ENSG00000172007.5, ENSG00000172071.10, ENSG00000172116.20, ENSG00000172197.10, ENSG00000172215.5, ENSG00000172243.16, ENSG00000172292.13, ENSG00000172322.12, ENSG00000172403.9, ENSG00000172469.13, ENSG00000172575.10, ENSG00000172578.10, ENSG00000172673.9, ENSG00000172724.10, ENSG00000172794.18, ENSG00000172795.14, ENSG00000172845.12, ENSG00000173145.10, ENSG00000173198.5, ENSG00000173200.11, ENSG00000173208.3, ENSG00000173221.12, ENSG00000173281.4, ENSG00000173369.14, ENSG00000173372.15, ENSG00000173391.7, ENSG00000173451.5, ENSG00000173578.7, ENSG00000173585.14, ENSG00000173626.8, ENSG00000173706.11, ENSG00000173757.8, ENSG00000173762.6, ENSG00000173889.14, ENSG00000174004.5, ENSG00000174123.9, ENSG00000174125.6, ENSG00000174197.15, ENSG00000174255.6, ENSG00000174485.13, ENSG00000174500.11, ENSG00000174600.12, ENSG00000174718.10, ENSG00000174799.9, ENSG00000174837.13, ENSG00000174885.11, ENSG00000174944.7, ENSG00000174946.6, ENSG00000175463.10, ENSG00000175489.9, ENSG00000175538.9, ENSG00000175841.8, ENSG00000175857.7, ENSG00000176160.8, ENSG00000176371.12, ENSG00000176907.4, ENSG00000176986.13, ENSG00000177076.5, ENSG00000177272.8, ENSG00000177311.9, ENSG00000177374.11, ENSG00000177455.10, ENSG00000177575.11, ENSG00000177590.7, ENSG00000177688.6, ENSG00000177721.4, ENSG00000177885.12, ENSG00000178175.10, ENSG00000178199.12, ENSG00000178343.4, ENSG00000178562.16, ENSG00000178789.7, ENSG00000179144.4, ENSG00000179163.11, ENSG00000179456.10, ENSG00000179715.11, ENSG00000179833.4, ENSG00000179840.5, ENSG00000179841.8, ENSG00000179921.13, ENSG00000179934.6, ENSG00000180061.8, ENSG00000180096.10, ENSG00000180139.11, ENSG00000180353.9, ENSG00000180448.9, ENSG00000180644.6, ENSG00000180884.9, ENSG00000181036.12, ENSG00000181631.6, ENSG00000181744.7, ENSG00000181804.13, ENSG00000181847.10, ENSG00000182022.16, ENSG00000182134.14, ENSG00000182162.8, ENSG00000182183.13, ENSG00000182463.14, ENSG00000182487.11, ENSG00000182568.15, ENSG00000182578.12, ENSG00000182866.15, ENSG00000182919.13, ENSG00000183023.17, ENSG00000183508.4, ENSG00000183542.5, ENSG00000183765.19, ENSG00000183801.6, ENSG00000183807.7, ENSG00000183813.6, ENSG00000184117.10, ENSG00000184156.14, ENSG00000184293.6, ENSG00000184619.3, ENSG00000184682.5, ENSG00000184922.12, ENSG00000185070.9, ENSG00000185245.7, ENSG00000185261.12, ENSG00000185271.6, ENSG00000185482.6, ENSG00000185650.9, ENSG00000185669.5, ENSG00000185739.12, ENSG00000185811.15, ENSG00000185900.8, ENSG00000185905.3, ENSG00000185947.13, ENSG00000186063.11, ENSG00000186074.17, ENSG00000186152.6, ENSG00000186198.3, ENSG00000186479.4, ENSG00000186517.12, ENSG00000186583.10, ENSG00000186635.13, ENSG00000186766.7, ENSG00000186818.11, ENSG00000186827.9, ENSG00000187037.7, ENSG00000187116.12, ENSG00000187210.11, ENSG00000187474.4, ENSG00000187513.8, ENSG00000187554.10, ENSG00000187653.11, ENSG00000187764.10, ENSG00000187796.12, ENSG00000187808.4, ENSG00000187862.10, ENSG00000188033.8, ENSG00000188107.12, ENSG00000188263.9, ENSG00000188389.9, ENSG00000188404.7, ENSG00000188559.12, ENSG00000188641.11, ENSG00000188820.11, ENSG00000188848.14, ENSG00000189144.12, ENSG00000189233.10, ENSG00000189350.11, ENSG00000196159.10, ENSG00000196209.11, ENSG00000196329.9, ENSG00000196371.3, ENSG00000196405.11, ENSG00000196468.7, ENSG00000196498.12, ENSG00000196504.14, ENSG00000196505.9, ENSG00000196664.4, ENSG00000196684.11, ENSG00000196839.11, ENSG00000196865.4, ENSG00000196911.8, ENSG00000196950.12, ENSG00000197142.9, ENSG00000197258.5, ENSG00000197272.2, ENSG00000197323.9, ENSG00000197405.6, ENSG00000197471.10, ENSG00000197548.11, ENSG00000197629.5, ENSG00000197872.10, ENSG00000197880.7, ENSG00000197943.8, ENSG00000197992.5, ENSG00000198075.8,

ENSG00000198178.9, ENSG00000198246.7, ENSG00000198286.8, ENSG00000198369.8, ENSG00000198399.13, ENSG00000198420.8, ENSG00000198586.12, ENSG00000198589.9, ENSG00000198624.11, ENSG00000198707.13, ENSG00000198771.9, ENSG00000198785.4, ENSG00000198821.9, ENSG00000198833.6, ENSG00000198851.8, ENSG00000198873.11, ENSG00000198879.10, ENSG00000198890.7, ENSG00000198900.5, ENSG00000198919.11, ENSG00000198924.6, ENSG00000198945.6, ENSG00000198959.10, ENSG00000203497.2, ENSG00000203710.9, ENSG00000203747.8, ENSG00000204131.7, ENSG00000204136.9, ENSG00000204161.12, ENSG00000204406.10, ENSG00000204472.11, ENSG00000204475.8, ENSG00000204482.9, ENSG00000204577.10, ENSG00000204745.3, ENSG00000204872.3, ENSG00000205089.6, ENSG00000205302.5, ENSG00000205436.6, ENSG00000205537.2, ENSG00000205683.10, ENSG00000205744.8, ENSG00000205784.2, ENSG00000205809.8, ENSG00000205810.7, ENSG00000205885.6, ENSG00000205930.7, ENSG00000211592.5, ENSG00000211593.2, ENSG00000211632.3, ENSG00000211669.2, ENSG00000211677.2, ENSG00000211688.1, ENSG00000211689.5, ENSG00000211694.2, ENSG00000211695.2, ENSG00000211698.2, ENSG00000211710.3, ENSG00000211713.3, ENSG00000211714.3, ENSG00000211716.2, ENSG00000211720.3, ENSG00000211724.3, ENSG00000211734.3, ENSG00000211746.3, ENSG00000211751.6, ENSG00000211752.3, ENSG00000211753.3, ENSG00000211764.1, ENSG00000211765.1, ENSG00000211766.1, ENSG00000211767.1, ENSG00000211768.1, ENSG00000211771.1, ENSG00000211772.7, ENSG00000211776.2, ENSG00000211778.2, ENSG00000211779.3, ENSG00000211780.3, ENSG00000211785.1, ENSG00000211786.3, ENSG00000211787.1, ENSG00000211788.2, ENSG00000211789.2, ENSG00000211790.2, ENSG00000211792.2, ENSG00000211793.2, ENSG00000211794.3, ENSG00000211795.3, ENSG00000211796.1, ENSG00000211797.2, ENSG00000211799.3, ENSG00000211801.3, ENSG00000211803.2, ENSG00000211804.3, ENSG00000211805.1, ENSG00000211806.2, ENSG00000211807.3, ENSG00000211809.2, ENSG00000211810.3, ENSG00000211814.1, ENSG00000211816.2, ENSG00000211818.1, ENSG00000211820.1, ENSG00000211821.2, ENSG00000211850.1, ENSG00000211873.1, ENSG00000211875.1, ENSG00000211876.1, ENSG00000211878.1, ENSG00000211879.1, ENSG00000211882.1, ENSG00000211885.1, ENSG00000211886.1, ENSG00000211896.5, ENSG00000211911.1, ENSG00000211949.3, ENSG00000211955.2, ENSG00000211959.2, ENSG00000211962.2, ENSG00000211972.2, ENSG00000213062.4, ENSG00000213203.2, ENSG00000213262.3, ENSG00000213809.7, ENSG00000213876.4, ENSG00000214077.4, ENSG00000214212.7, ENSG00000214269.3, ENSG00000215571.5, ENSG00000215910.6, ENSG00000216490.3, ENSG00000217258.2, ENSG00000217527.1, ENSG00000217643.1, ENSG00000220008.3, ENSG00000221043.2, ENSG00000221535.1, ENSG00000223459.5, ENSG00000223466.1, ENSG00000223552.1, ENSG00000223612.3, ENSG00000223750.1, ENSG00000223946.1, ENSG00000223969.4, ENSG00000224137.1, ENSG00000224220.1, ENSG00000224383.6, ENSG00000224460.1, ENSG00000224675.1, ENSG00000224875.2, ENSG00000225079.2, ENSG00000225205.4, ENSG00000225234.1, ENSG00000225325.1, ENSG00000225422.4, ENSG00000225460.1, ENSG00000225490.1, ENSG00000225731.1, ENSG00000225825.1, ENSG00000225885.5, ENSG00000225938.1, ENSG00000225974.1, ENSG00000226004.1, ENSG00000226423.1, ENSG00000226539.1, ENSG00000226660.2, ENSG00000226751.2, ENSG00000226777.6, ENSG00000226979.7, ENSG00000227007.1, ENSG00000227032.1, ENSG00000227145.1, ENSG00000227155.6, ENSG00000227191.5, ENSG00000227217.1, ENSG00000227295.2, ENSG00000227345.7, ENSG00000227449.7, ENSG00000227470.1, ENSG00000227507.2, ENSG00000227508.5, ENSG00000227531.1, ENSG00000227550.2, ENSG00000227678.6, ENSG00000227776.1, ENSG00000228005.1, ENSG00000228427.1, ENSG00000228763.1, ENSG00000228800.1, ENSG00000228804.4, ENSG00000228839.4, ENSG00000228986.1, ENSG00000229092.2, ENSG00000229153.4, ENSG00000229191.1, ENSG00000229228.1, ENSG00000229425.1, ENSG00000229473.2, ENSG00000229590.3, ENSG00000229613.1, ENSG00000229754.1, ENSG00000229816.1, ENSG00000229939.1, ENSG00000230006.6, ENSG00000230099.2, ENSG00000230107.1, ENSG00000230138.1, ENSG00000230155.5, ENSG00000230390.1, ENSG00000230499.1, ENSG00000230530.1, ENSG00000230709.1, ENSG00000230838.1, ENSG00000231105.1, ENSG00000231123.1, ENSG00000231128.4, ENSG00000231231.4, ENSG00000231241.1, ENSG00000231265.1, ENSG00000231435.1, ENSG00000231621.1, ENSG00000231690.2, ENSG00000231964.1, ENSG00000232208.2, ENSG00000232334.1, ENSG00000232613.5, ENSG00000232628.4, ENSG00000232698.1, ENSG00000232869.2, ENSG00000232884.6, ENSG00000233038.4, ENSG00000233093.4, ENSG00000233306.2, ENSG00000233387.1, ENSG00000233521.4, ENSG00000233673.5, ENSG00000233922.2, ENSG00000234142.1, ENSG00000234174.1, ENSG00000234184.4, ENSG00000234332.1, ENSG00000234389.1, ENSG00000234515.1, ENSG00000234568.3, ENSG00000234663.4, ENSG00000234816.2, ENSG00000235052.1, ENSG00000235151.1, ENSG00000235304.1, ENSG00000235419.4, ENSG00000235499.1, ENSG00000235532.1, ENSG00000235568.5, ENSG00000235586.1, ENSG00000235636.1, ENSG00000235659.1, ENSG00000235802.1, ENSG00000235831.5, ENSG00000236213.1, ENSG00000236278.2, ENSG00000236456.1, ENSG00000236469.1, ENSG00000236525.1, ENSG00000236846.1, ENSG00000236876.3, ENSG00000236911.5, ENSG00000236935.1, ENSG00000237254.2, ENSG00000237286.1, ENSG00000237470.3, ENSG00000237484.5, ENSG00000237513.1, ENSG00000237522.1, ENSG00000237604.1, ENSG00000237638.1, ENSG00000237702.2, ENSG00000237807.3, ENSG00000237914.4, ENSG00000237943.5, ENSG00000237955.1, ENSG00000237980.1, ENSG00000238171.1, ENSG00000238241.1, ENSG00000238290.1, ENSG00000239213.4, ENSG00000239281.2, ENSG00000239636.1, ENSG00000239941.1, ENSG00000239961.2, ENSG00000239964.3, ENSG00000239998.4, ENSG00000240143.1, ENSG00000240219.1, ENSG00000240292.1, ENSG00000240487.1, ENSG00000240505.7, ENSG00000240535.7, ENSG00000240654.5, ENSG00000240787.1, ENSG00000240891.5, ENSG00000240954.1, ENSG00000241106.5, ENSG00000241134.3, ENSG00000241158.4, ENSG00000241163.6, ENSG00000241351.2, ENSG00000241399.5, ENSG00000241490.1, ENSG00000241560.4, ENSG00000241717.1, ENSG00000241738.1, ENSG00000241962.8, ENSG00000242048.3, ENSG00000242258.1, ENSG00000242324.1, ENSG00000242574.7, ENSG00000242598.1, ENSG00000242736.1, ENSG00000243156.6, ENSG00000243232.4, ENSG00000243238.1, ENSG00000243544.3, ENSG00000243836.4, ENSG00000244227.4, ENSG00000244255.4, ENSG00000244273.1, ENSG00000244482.8, ENSG00000244661.1, ENSG00000244720.1, ENSG00000244968.5, ENSG00000245164.5, ENSG00000245648.1, ENSG00000245954.5, ENSG00000246084.2, ENSG00000246582.2, ENSG00000247199.3, ENSG00000247774.5, ENSG00000248383.4, ENSG00000248441.5, ENSG00000248571.1, ENSG00000248971.2, ENSG00000248996.1, ENSG00000249096.5, ENSG00000249129.1, ENSG00000249141.1, ENSG00000249334.1, ENSG00000249388.1, ENSG00000249437.6, ENSG00000249454.1, ENSG00000249667.1, ENSG00000249669.6, ENSG00000249835.2, ENSG00000249978.1, ENSG00000250050.1,

ENSG00000250541.1, ENSG00000250629.1, ENSG00000250654.6, ENSG00000250687.5, ENSG00000250722.4, ENSG00000251009.2, ENSG00000251131.1, ENSG00000251301.5, ENSG00000251332.1, ENSG00000251363.2, ENSG00000251664.3, ENSG00000251922.1, ENSG00000251962.1, ENSG00000252503.1, ENSG00000253361.1, ENSG00000253409.1, ENSG00000253535.4, ENSG00000253647.1, ENSG00000253686.1, ENSG00000253690.1, ENSG00000253701.2, ENSG00000253755.1, ENSG00000253822.1, ENSG00000253837.1, ENSG00000253930.1, ENSG00000253957.1, ENSG00000254041.1, ENSG00000254100.1, ENSG00000254167.1, ENSG00000254198.1, ENSG00000254340.1, ENSG00000254415.3, ENSG00000254760.1, ENSG00000254802.1, ENSG00000254838.5, ENSG00000254887.1, ENSG00000254911.3, ENSG00000254959.5, ENSG00000255090.4, ENSG00000255163.1, ENSG00000255197.4, ENSG00000255340.1, ENSG00000255422.1, ENSG00000255441.1, ENSG00000255569.1, ENSG00000255733.4, ENSG00000255819.5, ENSG00000255833.1, ENSG00000255882.1, ENSG00000256262.1, ENSG00000256540.1, ENSG00000257093.5, ENSG00000257221.1, ENSG00000257315.1, ENSG00000257594.3, ENSG00000257894.2, ENSG00000257906.1, ENSG00000257924.1, ENSG00000258086.1, ENSG00000258181.1, ENSG00000258511.1, ENSG00000258546.1, ENSG00000258810.1, ENSG00000258867.4, ENSG00000258878.1, ENSG00000258926.1, ENSG00000259004.1, ENSG00000259005.1, ENSG00000259124.1, ENSG00000259436.1, ENSG00000259628.1, ENSG00000259772.5, ENSG00000259834.1, ENSG00000259847.1, ENSG00000260228.4, ENSG00000260244.1, ENSG00000260314.2, ENSG00000260496.3, ENSG00000260517.2, ENSG00000260719.1, ENSG00000260828.1, ENSG00000260861.5, ENSG00000261208.1, ENSG00000261218.4, ENSG00000261269.1, ENSG00000261371.4, ENSG00000261416.1, ENSG00000261471.1, ENSG00000261644.1, ENSG00000261757.1, ENSG00000262039.1, ENSG00000262097.1, ENSG00000262151.1, ENSG00000262823.1, ENSG00000263264.1, ENSG00000263413.2, ENSG00000263809.1, ENSG00000264188.1, ENSG00000264219.1, ENSG00000264773.1, ENSG00000264781.1, ENSG00000264869.1, ENSG00000265148.4, ENSG00000265517.1, ENSG00000265612.1, ENSG00000265714.1, ENSG00000265719.1, ENSG00000265975.1, ENSG00000266094.5, ENSG00000266283.1, ENSG00000266389.1, ENSG00000266750.1, ENSG00000266804.1, ENSG00000267045.1, ENSG00000267364.1, ENSG00000267653.1, ENSG00000267654.1, ENSG00000267764.1, ENSG00000268027.4, ENSG00000268041.1, ENSG00000268201.1, ENSG00000268510.1, ENSG00000268861.4, ENSG00000269220.1, ENSG00000269404.5, ENSG00000269800.1, ENSG00000269904.2, ENSG00000269919.1, ENSG00000269937.1, ENSG00000269967.1, ENSG00000270547.4, ENSG00000270550.1, ENSG00000270661.1, ENSG00000271680.1, ENSG00000271779.1, ENSG00000271820.1, ENSG00000272053.1, ENSG00000272211.1, ENSG00000272256.1, ENSG00000272382.1, ENSG00000272477.1, ENSG00000272498.1, ENSG00000272563.1, ENSG00000272567.1, ENSG00000272763.1, ENSG00000272886.4, ENSG00000272908.1, ENSG00000272917.1, ENSG00000273107.1, ENSG00000273123.1, ENSG00000273172.1, ENSG00000273341.1, ENSG00000273348.1, ENSG00000273433.1, ENSG00000273445.1, ENSG00000273669.1, ENSG00000273837.1, ENSG00000273855.1, ENSG00000273923.1, ENSG00000274008.1, ENSG00000274128.1, ENSG00000274134.1, ENSG00000274172.1, ENSG00000274752.1, ENSG00000274961.1, ENSG00000275052.3, ENSG00000275158.1, ENSG00000275302.1, ENSG00000275743.1, ENSG00000275772.1, ENSG00000276231.3, ENSG00000276317.1, ENSG00000276334.1, ENSG00000276405.1, ENSG00000276454.1, ENSG00000276557.1, ENSG00000276819.1, ENSG00000276842.1, ENSG00000276961.1, ENSG00000276980.1, ENSG00000277030.1, ENSG00000277117.3, ENSG00000277734.3, ENSG00000277855.1, ENSG00000277882.1, ENSG00000278030.1, ENSG00000279078.1, ENSG00000279082.2, ENSG00000279192.1, ENSG00000279311.1, ENSG00000279380.1, ENSG00000279406.1, ENSG00000279481.1, ENSG00000279541.1, ENSG00000279631.1, ENSG00000280008.1, ENSG00000280014.1, ENSG00000280143.1, ENSG00000280194.1, ENSG00000280202.1, ENSG00000280304.1, ENSG00000280551.1, ENSG00000280734.1, ENSG00000281103.1, ENSG00000281741.1

Top 15 Pathway of Genes for selected miRNA, mRNA and Methylation Markers on Step 3

Category	Term	Count	LT	PH	PT	%	P-Value	Fold Enrichment	Bonferroni	Benjamini	FDR
KEGG_PATHWAY	Chemokine signaling pathway (*)	84	1630	186	6879	1.9	1.60E-10	1.9	4.80E-08	1.60E-08	2.20E-07
KEGG_PATHWAY	Cytokine-cytokine receptor interaction	83	1630	243	6879	1.9	1.90E-04	1.4	5.50E-02	4.70E-03	2.50E-01
KEGG_PATHWAY	Endocytosis	83	1630	241	6879	1.9	1.40E-04	1.5	4.00E-02	4.10E-03	1.90E-01
KEGG_PATHWAY	Focal adhesion	82	1630	206	6879	1.9	2.80E-07	1.7	8.30E-05	1.00E-05	3.70E-04
KEGG_PATHWAY	HTLV-I infection	103	1630	254	6879	2.4	2.00E-09	1.7	6.00E-07	1.20E-07	2.70E-06
KEGG_PATHWAY	MAPK signaling pathway	84	1630	253	6879	1.9	4.90E-04	1.4	1.30E-01	1.00E-02	6.50E-01
KEGG_PATHWAY	Osteoclast differentiation (*)	72	1630	131	6879	1.7	2.90E-14	2.3	8.60E-12	8.60E-12	3.90E-11
KEGG_PATHWAY	Pathways in cancer	155	1630	393	6879	3.6	1.20E-12	1.7	3.60E-10	1.80E-10	1.60E-09
KEGG_PATHWAY	PI3K-Akt signaling pathway	116	1630	345	6879	2.7	1.90E-05	1.4	5.60E-03	6.30E-04	2.60E-02
KEGG_PATHWAY	Proteoglycans in cancer	83	1630	200	6879	1.9	2.70E-08	1.8	7.90E-06	1.30E-06	3.60E-05
REACTOME_PATHWAY	R-HSA-983168	93	2158	308	9075	2.2	7.30E-03	1.3	1.00E+00	9.90E-01	1.10E+01
KEGG_PATHWAY	Rap1 signaling pathway (*)	91	1630	210	6879	2.1	3.70E-10	1.8	1.10E-07	2.70E-08	5.00E-07
KEGG_PATHWAY	Ras signaling pathway	91	1630	226	6879	2.1	3.00E-08	1.7	8.80E-06	1.30E-06	4.00E-05
KEGG_PATHWAY	Regulation of actin cytoskeleton	74	1630	210	6879	1.7	1.50E-04	1.5	4.40E-02	4.10E-03	2.00E-01
KEGG_PATHWAY	Viral carcinogenesis	71	1630	205	6879	1.6	3.80E-04	1.5	1.00E-01	8.50E-03	5.00E-01

Top 15 Pathway of Genes for selected miRNA, mRNA Markers on Step 2

Category	Term	Count	LT	PH	PT	%	P-Value	Fold Enrichment	Bonferroni	Benjamini	FDR
EC_NUMBER	6.3.2.-	137	2506	205	4250	1.2	1.60E-02	1.1	1.00E+00	1.00E+00	2.20E+01
KEGG_PATHWAY	Chemokine signaling pathway	141	3958	186	6879	1.3	2.40E-07	1.3	7.30E-05	1.00E-05	3.30E-04
KEGG_PATHWAY	Endocytosis	187	3958	241	6879	1.7	6.30E-11	1.3	1.90E-08	6.20E-09	8.40E-08
KEGG_PATHWAY	Focal adhesion	152	3958	206	6879	1.4	1.40E-06	1.3	4.10E-04	5.10E-05	1.80E-03
KEGG_PATHWAY	HTLV-I infection	201	3958	254	6879	1.8	3.60E-13	1.4	1.10E-10	5.30E-11	4.70E-10
KEGG_PATHWAY	MAPK signaling pathway	195	3958	253	6879	1.7	6.70E-11	1.3	2.00E-08	5.00E-09	9.00E-08
KEGG_PATHWAY	Pathways in cancer	302	3958	393	6879	2.7	3.10E-16	1.3	1.00E-13	1.00E-13	4.40E-13
KEGG_PATHWAY	PI3K-Akt signaling pathway	238	3958	345	6879	2.1	8.00E-06	1.2	2.40E-03	2.40E-04	1.10E-02
KEGG_PATHWAY	Proteoglycans in cancer	157	3958	200	6879	1.4	5.70E-10	1.4	1.70E-07	3.40E-08	7.50E-07
REACTOME_PATHWAY	R-HSA-212436	234	5317	358	9075	2.1	6.00E-03	1.1	1.00E+00	9.90E-01	9.40E+00
REACTOME_PATHWAY	R-HSA-983168	215	5317	308	9075	1.9	3.80E-05	1.2	5.30E-02	5.30E-02	6.30E-02
KEGG_PATHWAY	Rap1 signaling pathway	153	3958	210	6879	1.4	4.30E-06	1.3	1.30E-03	1.40E-04	5.80E-03
KEGG_PATHWAY	Ras signaling pathway	168	3958	226	6879	1.5	1.50E-07	1.3	4.50E-05	7.50E-06	2.00E-04
KEGG_PATHWAY	Regulation of actin cytoskeleton	151	3958	210	6879	1.4	1.70E-05	1.2	5.00E-03	4.50E-04	2.20E-02
KEGG_PATHWAY	Viral carcinogenesis	142	3958	205	6879	1.3	5.10E-04	1.2	1.40E-01	1.30E-02	6.80E-01

Top 15 Pathway of Genes for selected miRNA Markers STEP-1

Category	Term	Count	LT	PH	PT	%	P-Value	Fold Enrichment	Bonferroni	Benjamini	FDR
KEGG_PATHWAY	Pathways in cancer	125	1183	393	6879	4, 0	4, 6E-13	1, 8	1, 3E-10	1, 3E-10	9, 6E-11
KEGG_PATHWAY	Proteoglycans in cancer	72	1183	200	6879	2, 3	2, 0E-10	2, 1	5, 7E-8	1, 9E-8	1, 4E-8
KEGG_PATHWAY	Hepatitis B	55	1183	145	6879	1, 7	4, 4E-9	2, 2	1, 3E-6	2, 1E-7	1, 5E-7
KEGG_PATHWAY	Focal adhesion	70	1183	206	6879	2, 2	6, 1E-9	2, 0	1, 8E-6	2, 5E-7	1, 8E-7
KEGG_PATHWAY	Rap1 signaling pathway	66	1183	210	6879	2, 1	4, 8E-7	1, 8	1, 4E-4	1, 1E-5	8, 2E-6

KEGG_PATHWAY	HTLV-I infection	76	1183	254	6879	2, 4	5, 1E-7	1, 7	1, 5E-4	1, 1E-5	8, 2E-6
KEGG_PATHWAY	Ras signaling pathway	67	1183	226	6879	2, 1	3, 8E-6	1, 7	1, 1E-3	5, 0E-5	3, 6E-5
KEGG_PATHWAY	Viral carcinogenesis	61	1183	205	6879	1, 9	9, 7E-6	1, 7	2, 8E-3	1, 1E-4	7, 8E-5
REACTOME_PATHWAY	R-HSA-212436	94	1599	358	9075	3, 0	3, 4E-5	1, 5	3, 8E-2	1, 7E-2	1, 7E-2
KEGG_PATHWAY	PI3K-Akt signaling pathway	88	1183	345	6879	2, 8	7, 0E-5	1, 5	2, 0E-2	6, 0E-4	4, 3E-4
KEGG_PATHWAY	Endocytosis	63	1183	241	6879	2, 0	4, 3E-4	1, 5	1, 2E-1	2, 7E-3	1, 9E-3
KEGG_PATHWAY	cAMP signaling pathway	53	1183	198	6879	1, 7	7, 4E-4	1, 6	1, 9E-1	4, 3E-3	3, 1E-3
KEGG_PATHWAY	MAPK signaling pathway	63	1183	253	6879	2, 0	1, 6E-3	1, 4	3, 8E-1	7, 8E-3	5, 6E-3
KEGG_PATHWAY	Regulation of actin cytoskeleton	54	1183	210	6879	1, 7	1, 8E-3	1, 5	4, 0E-1	8, 1E-3	5, 8E-3
REACTOME_PATHWAY	R-HSA-983168	74	1599	308	9075	2, 4	3, 6E-3	1, 4	9, 8E-1	1, 6E-1	1, 5E-1

frontiers
